# Enhanced blue phosphorescence in platinum acetylide complexes *via* a secondary heavy metal and anion-controlled aggregation†

**DOI:** 10.1039/d5sc00172b

**Published:** 2025-03-25

**Authors:** Vinh Q. Dang, Chenggang Jiang, Thomas S. Teets

**Affiliations:** a University of Houston, Department of Chemistry 3585 Cullen Blvd. Room 112 Houston TX 77204-5003 USA tteets@uh.edu

## Abstract

Organoplatinum compounds represent a promising class of blue-phosphorescent molecules for electroluminescent color displays. Much recent work has focused on decreasing the nonradiative rate constant (*k*_nr_) to improve the photoluminescence quantum yield (*Φ*_PL_) of these compounds, but in most cases small radiative rate constants (*k*_r_) lead to long excited-state lifetimes (*τ*) poorly suited for electroluminescence applications. In this work, we present an approach to increase *k*_r_ and *Φ*_PL_ in blue-phosphorescent platinum acetylide complexes with the general formula *cis*-[Pt(CN–R)_2_(C≡C–2-py)_2_] (CN–R is an alkyl isocyanide and C≡C–2-py is 2-pyridylacetylide). This method incorporates secondary heavy metals, Cu(i) or Ag(i), bound by the pyridyl moieties. We observe the formation of dimer complexes in the solid state due to noncovalent interactions between the secondary metal and the acetylide ligands, especially strong in the case of Cu(i). Incorporation of Cu(i) also erodes the desired blue-phosphorescence by introducing a low-lying metal-to-ligand charge transfer (^3^MLCT) state that dominates the observed phosphorescence. In the complexes bound to Ag(i), we find that phosphorescence profile is strongly dependent on the counteranion, which we propose is caused by different degrees of aggregation. With this insight, we show that coordination of AgBAr^F^_4_ (BAr^F^_4_^−^ = tetrakis[3,5-bis(trifluoromethyl)phenyl]borate), with a large noncoordinating counteranion, inhibits aggregation and results in a 4–8× increase in *k*_r_ and a 5–10× increase in *Φ*_PL_ while preserving a pure blue phosphorescence profile.

## Abstract 2

Pyridyl-substituted platinum acetylide complexes bind coinage metal cations, strongly influencing photoluminescence properties. Large counterions maintain the blue phosphorescence profile with large increases in radiative rate and quantum yield.

## Graphical abstract

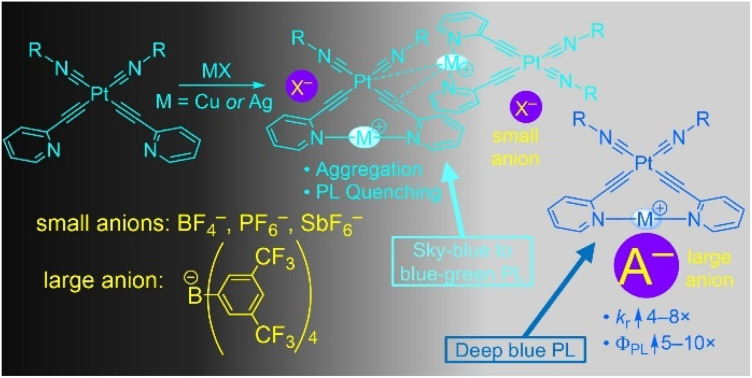

## Introduction

Electroluminescent devices convert electricity to light, and some categories, most prominently organic light-emitting diodes (OLEDs), use luminescent molecules to generate the light. These technologies have gained significant attention because of their outstanding performance in color display panels.^1^ OLEDs doped with phosphorescent transition-metal complexes have 100% theoretical internal efficiency, enabled by strong spin–orbit coupling (SOC) in the dopant, and can also offer superb color quality, light weight, high stability, and easy modification.^2–4^ Green and red OLEDs with phosphorescent dopants have been successfully commercialized, and significant progress has been made to produce blue OLEDs with high performance and long device lifetimes. Nevertheless, the lack of suitable phosphorescent dopants for blue OLEDs remains a challenge and limits their efficiency.^5^ As such, efforts to design blue-phosphorescent dopants with appropriate color profiles and high photoluminescence quantum yields are still at the forefront.^6,7^

**Fig. 1:**
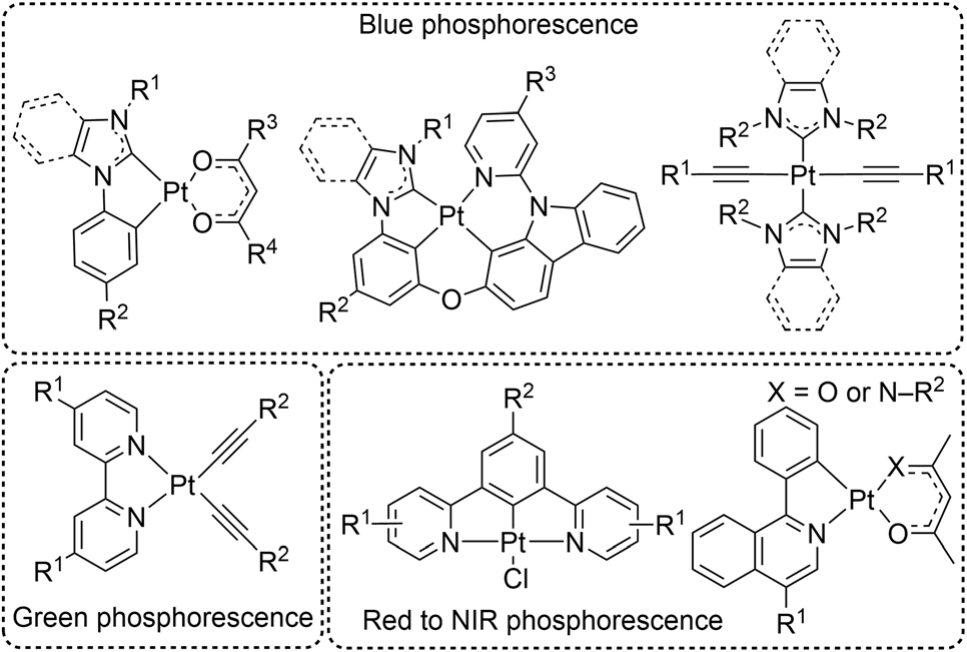
Structure of representative phosphorescent platinum complexes.

In general, cyclometalated iridium complexes have been the most prominent class of molecular phosphors for optoelectronic applications,^8^ including many recent fundamental and applied studies on blue-phosphorescent analogues.^9–17^ Alternatively, there are several classes of platinum complexes that present as promising candidates for accessing a variety of photoluminescence colors (Fig. 1). Cyclometalated platinum(ii) complexes, in which the cyclometalating ligand can be bidentate, tridentate, or even tetradentate, have been widely explored due to their high quantum yields and ability to fine-tune the emission color.^18–23^ Blue-phosphorescent analogues with bidentate^24–28^ or tetradentate^29–34^ cyclometalating ligands as well as platinum acetylide complexes^7,35–38^ with exclusively monodentate ligand sets have attracted significant fundamental interest and hold high potential for technological development. Some of these compounds exhibit excellent color purity in the deep blue region with impressive photoluminescence quantum yields (*Φ*_PL_), making them attractive for OLED applications.

Recent fundamental studies on blue-phosphorescent compounds have largely focused on reducing the nonradiative rate constant (*k*_nr_), which is particularly high in the blue region due to the thermal population of higher-lying, nonradiative ligand-field states, often referred to as metal-centered states (^3^MC), that reside near the emissive T_1_ state. A common strategy involves incorporating strong σ-donor ligands to destabilize the ^3^MC states, making them thermally inaccessible and thereby reducing the nonradiative rate constant. Some strong σ-donor ligands used for this purpose include acyclic diaminocarbene (ADC)^13,28,39–43^ and N-heterocyclic carbene (NHC)^6,9,10,35,44^ derivatives.

Although the above-mentioned approaches are effective at increasing *Φ*_PL_, a persistent challenge in blue-phosphorescent platinum complexes is that the radiative rate constants (*k*_r_) are inherently small, leading to long excited-state lifetimes (*τ*) that are unsuitable for some optoelectronic applications, OLEDs included. The main reason for the smaller *k*_r_ values in Pt(ii) complexes compared to Ir(iii) is the less effective SOC in the T_1_ states of Pt(ii) compounds, owing to the large separation between the highest-occupied 5d orbital (d_*z*^2^_) and the lower-lying 5dπ orbitals in the square-planar coordination environment.^19^ Therefore, a substantial fundamental challenge in the field of blue phosphorescence is to elucidate molecular design strategies that enhance spin–orbit coupling (SOC) and increase radiative rate constants in blue-phosphorescent platinum(ii) complexes. There are several strategies that incorporate secondary heavy metals into Ir(iii) or Pt(ii) complexes as a means of improving photophysical properties,^45–54^ oftentimes effective at increasing *k*_r_ in red-phosphorescent compounds but not previously applied to compounds luminescing in the blue region. Relatedly, there has been an attempt to apply a similar strategy to blue-phosphorescent Pt(ii) complexes *via* covalent attachment of secondary heavy atoms (Br), but in this case the bromine atoms made minimal contributions to the excited states and no significant improvements were realized.^55^

Platinum(ii) acetylide complexes have sharp phosphorescence profiles in the blue region, but like most organoplatinum complexes suffer from weak SOC and small *k*_r_ values, as in this case the T_1_ state is mainly a ^3^(π → π*) state localized on the acetylides with only minor contribution to the metal.^27,42,43,56^ Enhancing SOC and *k*_r_ is critical if this promising class of compounds is ever to become viable for practical optoelectronic applications. In this work, we introduce an approach to increase *k*_r_ in blue-phosphorescent platinum acetylide complexes by coordinating the coinage metals Cu(i) and Ag(i) to the 2-pyridyl acetylide ligands. We propose that these secondary heavy atoms may introduce additional metal orbital contributions to the emissive T_1_ state centered on the 2-pyridyl acetylide ligands, increasing SOC and improving phosphorescence metrics. Upon coordination of coinage metals, aggregation is observed in condensed phases *via* noncovalent interactions involving acetylide C≡C π electrons and/or the Pt(ii) center. With copper, this aggregation and the likely introduction of low-energy ^3^MLCT states involving Cu(i) shifts the phosphorescence out of the blue region, resulting in broad, long-wavelength PL. We find that the introduction of Ag(i) salts allows two layers of control over the photoluminescence properties – coordination of Ag(i) significantly increases *k*_r_*via* the secondary heavy-atom effect, while the size of the counterion controls the degree of aggregation and thus is responsible for the observed phosphorescence color profile. Combining these two insights and using AgBAr^F^_4_ as the Ag(i) source, we formulate two Pt(ii)–Ag(i) heterobimetallic complexes that maintain deep-blue phosphorescence with *Φ*_PL_ 5–10× higher and *k*_r_ 4–8× higher than the respective Pt(ii) acetylide complex in the absence of Ag(i).

## Results and discussion

### Synthesis

**Scheme 1:**
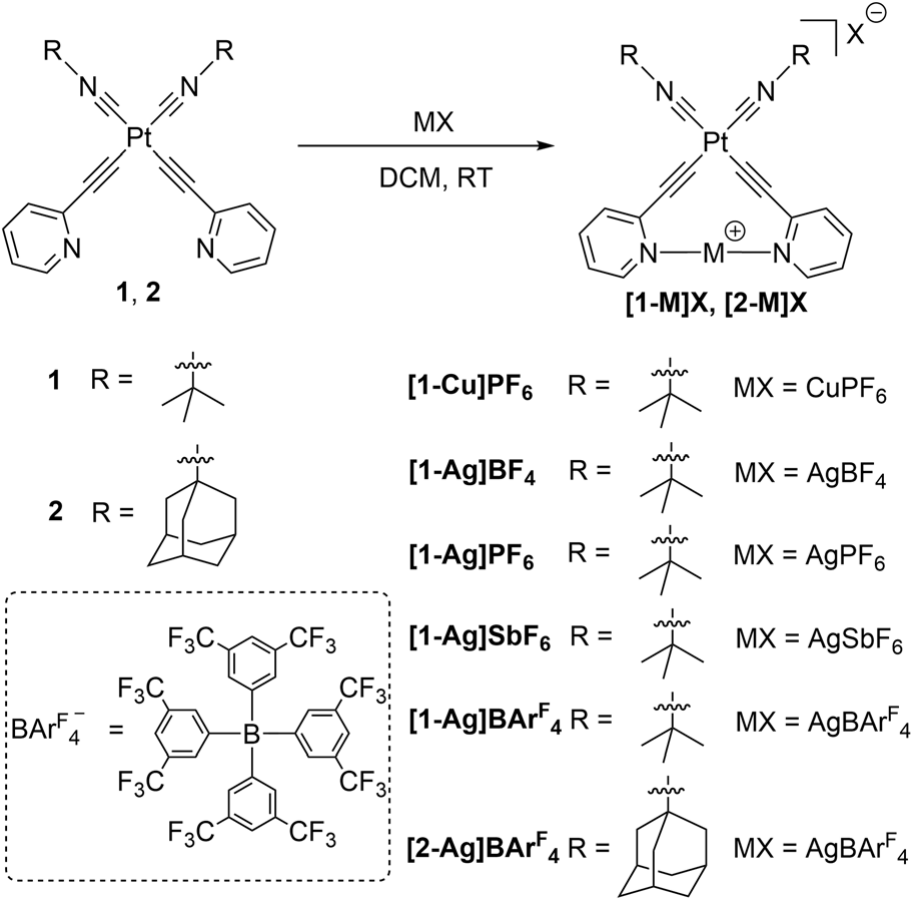
Synthesis of new bimetallic Pt–M complexes.

The synthetic procedure for incorporating coinage metal ions into pyridyl-substituted platinum acetylide complexes is presented in Scheme 1. Scheme S1 in the ESI† also summarizes the preparation of precursor complexes 1 and 2, which follows the general strategy developed by our group in previous works.^42,43^ Initially, the [Pt(COD)(C≡C–2-py)_2_] precursor, where COD is 1,5-cyclooctadiene and C≡C–2-py is 2-pyridylacetylide, was synthesized *via* ligand exchange between [Pt(COD)Cl_2_] and 2-ethynylpyridine in the presence of potassium *tert*-butoxide (KO^*t*^Bu). Then, isocyanide ligands were substituted onto [Pt(COD)(C≡C–2-py)_2_] to achieve the bis-isocyanide complexes with the general formula *cis*-[Pt(CN–R)_2_(C≡C–2-py)_2_], where R is *tert*-butyl (complex 1) or 1-adamantyl (complex 2). Finally, the coinage metals ions were introduced by adding the corresponding salts [Cu(NCMe)_4_]PF_6_ ([1-Cu]PF_6_), AgBF_4_ ([1-Ag]BF_4_), AgPF_6_ ([1-Ag]PF_6_), AgSbF_6_ ([1-Ag]SbF_6_), and AgBAr^F^_4_ ([1-Ag]BAr^F^_4_, [2-Ag]BAr^F^_4_) in CH_2_Cl_2_ solvent, where BAr^F^_4_^−^ is tetrakis[3,5-bis(trifluoromethyl)phenyl]borate. Due to the low solubility of these complexes in CH_2_Cl_2_ (except for the BAr^F^_4_^−^ analogues), in most cases the product quickly precipitated and was easily purified *via* filtration with good yield. The products [1-Cu]PF_6_, [1-Ag]BF_4_, and [1-Ag]PF_6_ are sparingly soluble in most organic solvents (*e.g.*, CH_2_Cl_2_, THF, and CH_3_Cl), but their good solubility in CH_3_CN makes it the best choice for solution characterization and photophysical experiments. The solubility of both [1-Ag]BAr^F^_4_ and [2-Ag]BAr^F^_4_ is much higher, and they can be readily dissolved in most polar organic solvents. Attempts to coordinate gold(i) to complex 1*via* metathesis of [1-Ag]PF_6_ with [Au(tht)Cl] (tht = tetrahydrothiophene) were unsuccessful.

### Structural characterization

The identity and purity of all the complexes were validated by NMR spectroscopy (Fig. S24–S45†). The ^1^H NMR spectra clearly show the presence of both isocyanide and acetylide ligands with a 1 : 1 ratio in all complexes. After introducing Cu(i) or Ag(i), the ^1^H NMR peaks in the aromatic region shift downfield due to the electron-withdrawing properties of the secondary metal cations. This shift is most pronounced for H^3^ (the pyridyl nitrogen is numbered as the 2-position, see Fig. S1†), which is proximal to the site of cation binding. On the other hand, the aromatic peak assigned to H^6^ has a smaller downfield shift (*ca.* 0.07 ppm) compared to other aromatic peaks, attributed to the meta orientation of this proton relative to the pyridyl nitrogen atom. These observed shifts of the ^1^H NMR peaks assigned to the pyridyl protons are consistent with the previous work of Erdélyi's group on bis(pyridine)silver(i) complexes.^57^ The upfield CH_3_ singlet in the *tert*-butyl isocyanide complexes is virtually unshifted upon metal ion binding. All of these observations suggest that Ag(i) and Cu(i) are chelated by the two 2-pyridyl acetylide ligands. Additionally, the counterions were verified by an appropriate combination of ^19^F, ^31^P{^1^H}, and ^11^B{^1^H} NMR. Furthermore, in complexes [1-Ag]BAr^F^_4_ and [2-Ag]BAr^F^_4_, besides ^11^B{^1^H} NMR and ^19^F NMR, the presence of the BAr^F^_4_^−^ anion was also confirmed *via*^1^H NMR, with the integration validating the 1 : 1 ratio of the counterion and the platinum acetylide. FT-IR spectra (Fig. S16–S23†) provide additional spectroscopic characterization of the isolated products. A single ṽ(C≡C) band (2070–2142 cm^−1^) was resolved in all complexes, but in all cases except [1-Cu]PF_6_ there are two distinct ṽ(C≡N) stretching bands (2206–2256 cm^−1^), consistent with the *cis* geometry and approximate *C*_2v_ symmetry of these complexes.

**Fig. 2:**
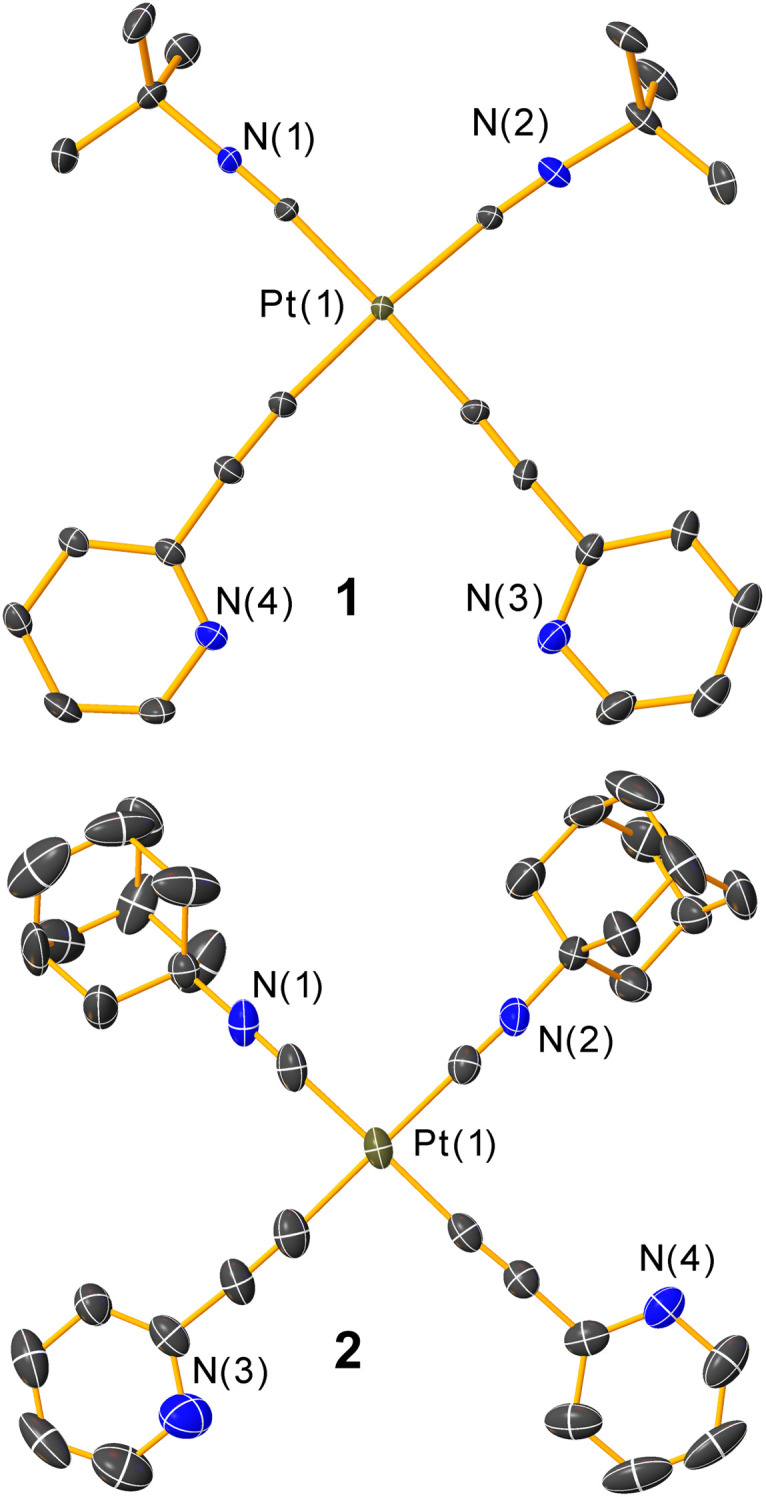
Molecular structures of complexes 1 and 2, determined by single-crystal X-ray diffraction. Thermal ellipsoids are shown at 50% probability level with hydrogen atoms omitted. In both cases, only one of the two crystallographically independent molecules is shown.

The solid-state structures of complexes 1, 2, [1-Cu]PF_6_, and [1-Ag]PF_6_ were determined by single-crystal X-ray diffraction. Molecular structures for the coinage-metal-free complexes 1 and 2 are shown in Fig. 2 with their crystallographic data summarized in Table S1.† In complexes 1 and 2, all four ligands are nearly coplanar with a *cis* configuration, resulting in approximate *C*_2v_ symmetry. The C≡C and C≡N internuclear distances are consistent with triple bonds and minimal backbonding, typical for related platinum(ii) acetylide complexes.^40–43^

**Fig. 3:**
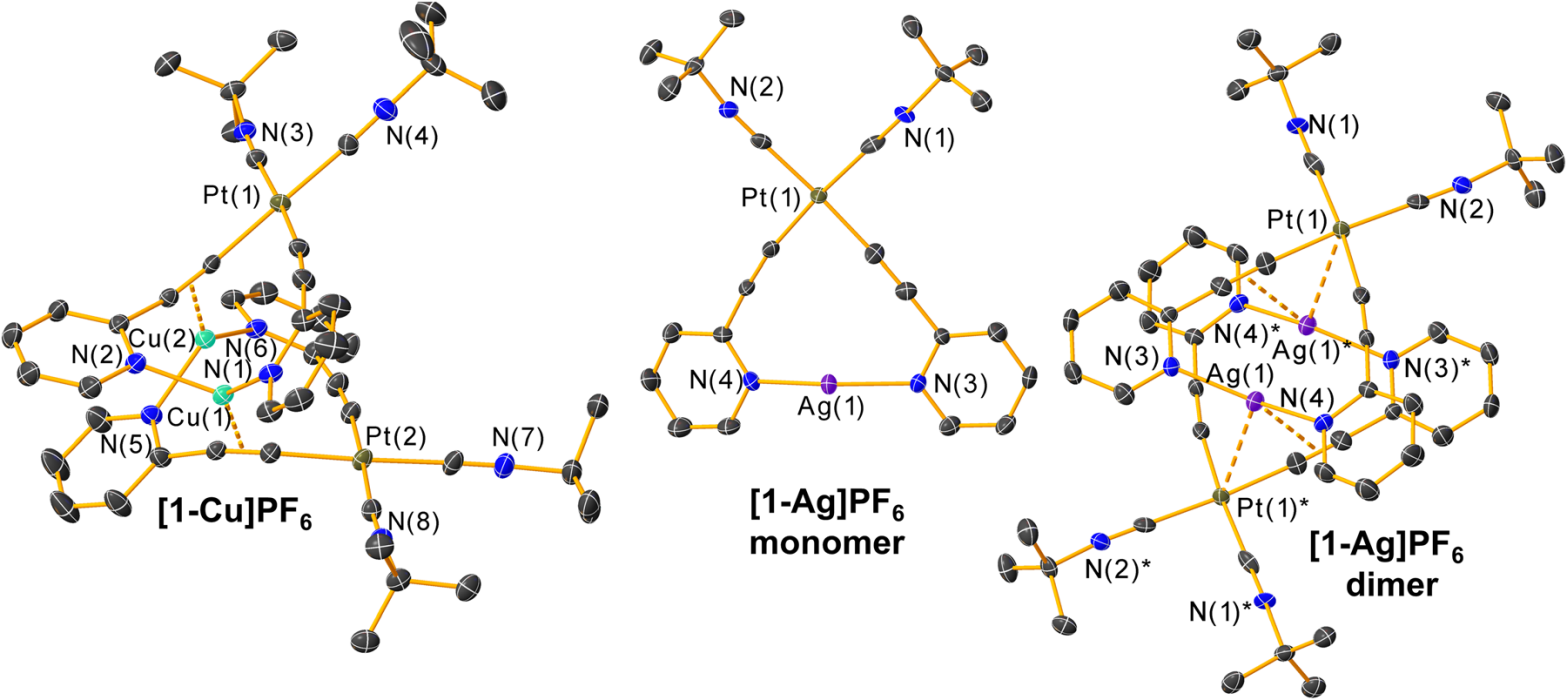
Molecular structures of complexes [1-Cu]PF_6_ and [1-Ag]PF_6_, determined by single-crystal X-ray diffraction. Thermal ellipsoids are shown at 50% probability level with hydrogen atoms, solvent molecules, and counterions omitted. For the structure of [1-Ag]PF_6_, the monomer structure that represents the asymmetric unit is shown, as well as the dimer that results when the second molecule is generated by crystallographic symmetry. In the dimer structure, labels marked with an asterisk (*) mark the atoms that are symmetry-generated.

The structures of [1-Cu]PF_6_ and [1-Ag]PF_6_ are shown in Fig. 3 with the data summarized in Table S2.† They confirm that the primary binding mode of the coinage metal cation involves coordination of two 2-pyridyl acetylide ligands, *i.e.*, chelation by the platinum bis-acetylide metalloligand. The monomer structure of [1-Ag]PF_6_ shown in Fig. 3 most clearly depicts that primary interaction. However, in both cases coordination of the coinage metal cation induces dimerization through secondary interactions; in [1-Cu]PF_6_ the asymmetric unit consists of the Pt_2_Cu_2_ dimer, whereas in [1-Ag]PF_6_ the second molecule in the dimer is generated *via* crystallographic symmetry. Fig. S5 and S6 in the ESI† show zoomed-in views of the tetrametallic cores of the dimer, to more easily visualize the secondary interactions that assemble the metal ions. In the copper(i) analogue, the two molecules in the dimer are in a twisted arrangement and held together by a bond between the Cu atom and the C≡C π electrons. The distances between the Cu atoms and the centroids of the C≡C bonds to which they coordinate are 1.93 and 1.94 Å, shorter than the Cu–N distances that span 1.98–2.02 Å. This results in each Cu(i) having a three-coordinate planar geometry. In the case of the Ag(i) analogue, a head-to-tail arrangement is observed and the stabilizing forces that drive dimer formation appear to be weaker. There is likewise an interaction involving the acetylide π electrons, but the distance in this case (C≡C centroid to Ag) is 2.78 Å, almost 1 Å longer than Cu. Stronger coordination of acetylide π electrons to Cu(i) *vs.* Ag(i) has also been observed in titanocene complexes.^58^ The interactions between the coinage metal cations and the acetylide π electrons slightly elongate the C≡C bonds, with the uncoordinated acetylide triple bonds in 1 averaging 1.193 Å, elongating by *ca.* 0.03 Å when Cu(i) or Ag(i) is incorporated. Along with elongating the C≡C bonds, the C–Pt–C bond angles involving the *cis*-oriented acetylide ligands contract by *ca.* 4–6° upon the incorporation of a coinage metal, suggesting a small “tweezer” effect where the pyridyl rings clamp down on the exogenous metal ion. In addition, a weak noncovalent Pt⋯Ag interaction of 3.11 Å is apparent in the crystal structure of [1-Ag]PF_6_. We do not see any evidence for cuprophilic (Cu⋯Cu) or argentophilic (Ag⋯Ag) interactions in the structures. In both cases, the shortest distance between two neighboring coinage metal ions is an intra-dimer contact of *ca.* 3.6 Å, significantly longer than the accepted limits for such metallophilic interactions.^59,60^

The observed dimerization that occurs in the solid state led us to investigate whether the noncovalent interactions responsible for dimerization would persist in solution for complex [1-Ag]PF_6_. Concentration-dependent ^1^H NMR spectra were recorded for complexes 1 and [1-Ag]PF_6_, presented in Fig. S1 and S2,† respectively. The ^1^H NMR spectrum of complex 1 is independent of concentration, over the range of 4 mg mL^−1^ to 20 mg mL^−1^. In contrast, the ^1^H NMR spectrum of complex [1-Ag]PF_6_ evolves over this same concentration range. The ^1^H NMR peak assigned to the CH_3_ groups of the isocyanide do not shift, which is consistent with crystallographic data that shows no interaction between the Ag(i) ion and the isocyanide ligands. In contrast, in the aromatic region, the peak assigned to H^6^ of the pyridyl acetylide, which is adjacent to the acetylide group, shifts upfield by 0.07 ppm when concentration increased from 4 mg mL^−1^ to 20 mg mL^−1^. The resonance for H^3^, adjacent to the pyridyl nitrogen, is minimally changed. These observations are consistent with aggregation through an Ag–alkyne interaction, which mainly impacts the H^6^ resonance that is *ortho* to the acetylide substituent.

Since most of the isolated platinum–silver complexes are only soluble in MeCN, we were concerned about the possibility of MeCN displacing Ag^+^ in solution. Thus, along with the concentration-dependent ^1^H NMR study described above, a titration experiment was conducted to investigate the behavior of complex 1 in the presence of varying amounts of AgPF_6_ in CD_3_CN solvent (Fig. S3†). When substoichiometric aliquots of AgPF_6_ stock solution (0.032 M) are added to the solution of complex 1 (0.009 M), a single set of ^1^H NMR aromatic peaks is observed, intermediate in chemical shift between those of complex 1 and [1-Ag]PF_6_. This indicates that the Ag(i) ion is labile, and that exchange is fast on the NMR timescale. When superstoichiometric amounts of AgPF_6_ are added to complex 1, the ^1^H NMR spectrum remains nearly identical to that of complex [1-Ag]PF_6_, showing that complex 1 is only able to quantitatively bind 1 equivalent of Ag(i) with no significant interaction of a second equivalent. To further investigate the potential lability of the silver cation in solution, variable-temperature ^1^H NMR of a mixture of complex 1 and AgPF_6_ was conducted over the temperature range of 25 °C to −35 °C (Fig. S4†). As the temperature decreases, most of peaks shift similarly to what occurs when the concentration is increased, except for the H^3^ peak which shifts downfield. This result suggests enhanced aggregation at low temperature, while the disparate behavior of the H^3^ peak may result from temperature-induced chemical shift changes. Notably, a single set of NMR peaks is observed throughout the experiment, indicating the fast silver exchange process over the entire temperature range. Finally, we also showed that [1-Ag]BF_4_ can be synthesized from [Ag(CH_3_CN)_4_]BF_4,_ and complex 1, suggesting that the pyridine nitrogen atoms can readily displace CH_3_CN from Ag^+^, as observed in some other coordination compounds.^61–63^ All of the above evidence is consistent with a thermodynamic preference for the silver cation to be bound by the platinum acetylide complex instead of MeCN, even when MeCN is the solvent.

### Photophysical properties

**Fig. 4:**
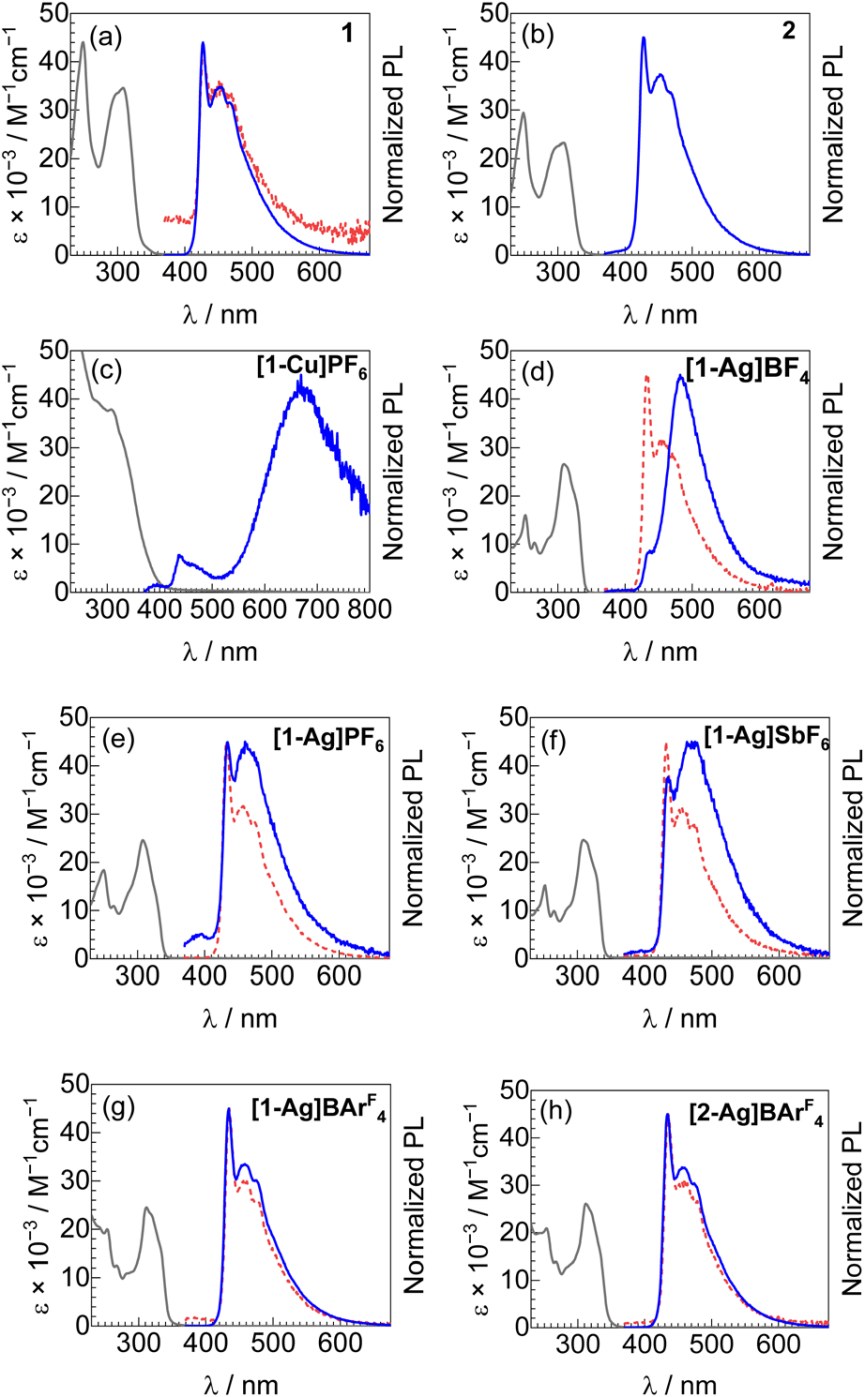
Overlaid UV-vis absorption and normalized photoluminescence (PL) spectra for (a) 1, (b) 2, (c) [1-Cu]PF_6_, (d) [1-Ag]B_46_, (e) [1-Ag]PF_6_, (f) [1-Ag]SbF_6_, (g) [1-Ag]BAr^F^_4_, and (h) [2-Ag]BAr^F^_4_. UV-vis absorption spectra (black solid lines) and PL spectra (red dashed lines) were both recorded in solution, using CH_2_Cl_2_ solvent for 1, [1-Ag]BAr^F^_4_, and [2-Ag]BAr^F^_4_, and CH_3_CN for the rest. PL spectra in 2 wt% PMMA films (blue solid lines) are also shown. Samples were excited at *λ* = 310 nm for all PL spectra. Due to the weak PL of complex 1 in solution, the signal-to-noise ratio is poor, and the baseline is not at zero.

UV-vis absorption and photoluminescence spectra of all complexes are shown in Fig. 4 and summarized in Tables 1 and S3.† There are no significant differences in the UV-vis absorption spectra of all complexes, except complex [1-Cu]PF_6_ for which absorption tails to longer wavelengths. Complexes 1, 2, and the silver-bound analogues show two strong absorption peaks in the 249–253 nm and 308–312 nm regions. According to previous works,^27,42,43,56^ these absorption bands can be primarily assigned as ^1^(π → π*) transitions of the acetylide ligands. In complex [1-Cu]PF_6_, the absorption spectrum is broad with a tail to near 500 nm. These features are tentatively ascribed to a ^1^MLCT transition involving the Cu(i) ion and the pyridyl acetylide ligands, possibly influenced by the strong aggregation that occurs in this complex (see above).

Complexes 1 and 2 exhibit similar photoluminescence spectra in PMMA films at 2 wt% with peak *λ*_max_ = 427 nm and 428 nm, respectively. These values are consistent with our previous works on bis-isocyanide platinum aryl acetylide complexes, minimally shifted in these pyridyl analogues relative to phenylacetylide analogues.^43^ The vibronic structure is also identical for both complexes suggesting that the triplet state mainly localizes on the pyridyl acetylide ligands. The photoluminescence quantum yields, measured in PMMA films at 2 wt%, are low in both complexes 1 (*Φ*_PL_ = 0.014) and 2 (*Φ*_PL_ = 0.024) with fast nonradiative rate constants (*k*_nr_ = 6.6 × 10^4^ s^−1^ for complex 1 and *k*_nr_ = 5.7 × 10^4^ s^−1^ for complex 2).

Introduction of copper(i) to complex 1 erodes the blue phosphorescence, with the photoluminescence spectrum of [1-Cu]PF_6_ in PMMA dominated by a broad band centered at 669 nm, albeit very weak (*Φ*_PL_ < 0.01), which we attribute to a ^3^MLCT state involving the redox-active Cu(i) ion and π* orbitals on the pyridyl acetylide ligands. It is also likely that this photoluminescence is influenced by the aggregation we observe in [1-Cu]PF_6_ (see Fig. 3), although given the absence of structural evidence (see above) we don't think cuprophilic interactions are responsible. Nevertheless, since Cu(i) binding obviates the targeted blue phosphorescence and results in very low quantum yields, we did not pursue a deeper understanding of the photoluminescence of this compound.

In the case of bimetallic Pt–Ag complexes ([1-Ag]X and [2-Ag]BAr^F^_4_), while the quantum yields are still low in solution (*Φ*_PL_ = 0.002–0.005), the luminescence in solution (Fig. 4d–h, overlaid in Fig. S7† and summarized in Table S3†) is intensified relative to 1 (weaker luminescence) and 2 (no luminescence in solution). As confirmation of this observation, we find that titration of AgPF_6_ into a solution of 1 results in the growth of new absorption bands attributed to the formation of [1-Ag]PF_6_, with a concomitant increase in photoluminescence intensity, which essentially ceases after 1 equivalent has been added (Fig. S8†).

Due to the nearly planar geometry of [1-Ag]PF_6_, we anticipated that aggregation modes akin to those observed crystallographically (Fig. 3), or others involving Pt⋯Pt and/or π stacking that are common in organoplatinum complexes, would increase in condensed media. These aggregation modes could deleteriously impact luminescence *via* aggregation-caused quenching (ACQ) or the introduction of lower-energy aggregated excited states that shift luminescence out of the blue region. We also hypothesized that the counterion would closely associate with the aggregated cations, and thus, the size of the counterion may play an important role in controlling aggregation and optimizing luminescence. To validate this hypothesis of anion-dependent aggregation, a series of bimetallic complexes with different counterions ([1-Ag]X, X = BF_4_^−^, PF_6_^−^, SbF_6_^−^, and BAr^F^_4_^−^) was investigated. In solution with concentrations of the samples spanning 1.5–6.6 × 10^−5^ M, identical photoluminescence spectra (Fig. S7†) are observed in all four of these complexes, consistent with aggregation not being significant in dilute fluid solutions. Conversely, a band to the red of the sharp *λ*_0–0_ peak, in the 450–500 nm region, grows in when these complexes are immobilized in PMMA films at 2 wt%. The appearance of this band is suggestive of aggregation in the films, and consistent with this notion, in all cases the intensity of the longer-wavelength band, relative to the sharp *λ*_0–0_ band, increases as the wt% of the complex in the PMMA film increases (Fig. S8†). We thus reasoned that the extent of aggregation, and thus the photoluminescence profile, could be controlled by the choice of counterion. The smallest counterion in the series in [1-Ag]BF_4_ results in a PL spectrum that is dominated by the broad band attributed to aggregation (482 nm, see Fig. 4d). When the size of the counterion increases (BF_4_^−^ < PF_6_^−^ < SbF_6_^−^ < BAr^F^_4_^−^), the luminescence arising from aggregation is significantly attenuated. Notably, when the largest counterion in the series is used in [1-Ag]BAr^F^_4_ and [2-Ag]BAr^F^_4_, the PL spectra in 2 wt% PMMA film are nearly identical to those in solution and are also minimally altered from those of the precursors 1 and 2, suggesting that aggregation is mostly suppressed.

Aggregation also has an influence on the observed photoluminescence quantum yield. In addition to concentration-dependent PL spectra in PMMA (Fig. S9†), quantum yields at increasing wt% loading were also recorded (Table S4†). In all complexes, including 1 and 2 which lack the Ag(i) ion, photoluminescence quantum yields decrease as the concentration in the film increases, indicating aggregation-caused quenching (ACQ) is at play. Thus, in the Pt–Ag complexes with smaller counterions, aggregation not only results in the growth of a new band at *ca.* 482 nm but also reduces the quantum yield. However, in the complexes with the largest counterion, [1-Ag]BAr^F^_4_ and [2-Ag]BAr^F^_4_, the bulky counterion not only allows a deep-blue phosphorescence profile to be maintained but also allows for much higher *Φ*_PL_ values. At the extremes, we report *Φ*_PL_ < 0.01 at 2 wt% loading for [1-Ag]BF_4_, the compound with the smallest counterion in the series, which increases dramatically to 0.14 in [1-Ag]BAr^F^_4_ at the same film loading.

Our initial hypothesis was the “secondary heavy-atom effect” introduced by the coordinated coinage metals could augment radiative rate constants in these compounds. This hypothesis is most clearly evaluated in the compounds 1, 2, [1-Ag]BAr^F^_4_, and [2-Ag]BAr^F^_4_, where the large counteranion suppresses effects caused by aggregation. In PMMA films at 2 wt%, the quantum yields of compounds [1-Ag]BAr^F^_4_ (*Φ*_PL_ = 0.14) and [2-Ag]BAr^F^_4_ (*Φ*_PL_ = 0.12) are a factor of 5–10× higher than those of compounds 1 (*Φ*_PL_ = 0.014) and 2 (*Φ*_PL_ = 0.024). The large increase in quantum yield is mainly caused by the enhancement of *k*_r_ values by a factor 4–8× higher. Binding the Ag(i) ion in [1-Ag]BAr^F^_4_ and [2-Ag]BAr^F^_4_ has much smaller effect on the nonradiative rate constant, which is slightly suppressed by ∼15–25%.

**Fig. 5:**
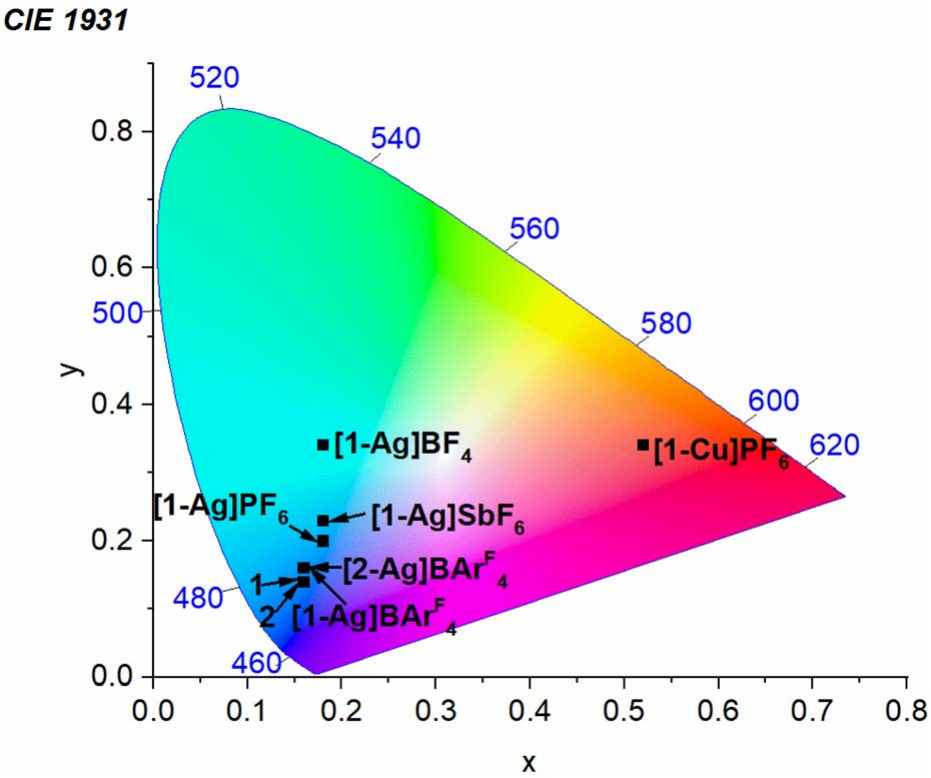
Chromaticity diagram showing the (CIE*x*, CIE*y*) coordinates of new platinum complexes, determined from photoluminescence spectra recorded in PMMA films at 2 wt%.

To further characterize the effects of the coinage metal cations on the luminescence profile, CIE coordinates were determined from the photoluminescence spectra recorded in PMMA films at 2 wt% (Table 1 and Fig. 5). Complexes 1 and 2 exhibit pure blue luminescence, with CIE coordinates of (0.16, 0.14). As described above, coordination of Cu(i) shifts the luminescence into the red region. In Pt–Ag complexes with significant aggregation, a substantial change in color profile is observed, with the PL shifting into the sky-blue ([1-Ag]PF_6_ and [1-Ag]SbF_6_) or blue-green regions ([1-Ag]BF_4_). In contrast, with AgBAr^F^_4_ there is a slight red shift in *λ*_0–0_, but suppression of aggregation allows the photoluminescence profile to remain in the pure blue region. There is only a small change in CIE*y*, which increases from 0.14 to 0.16 in the AgBAr^F^_4_ complexes. Thus, the addition of AgBAr^F^_4_ preserves the desired color profile while also augmenting *k*_r_ and *Φ*_PL_.

**Table 1:** Summary of UV-vis absorption and photoluminescence data

Complex	UV-vis absorption^a^	Photoluminescence						
	*λ*_max_/nm (*ε* × 10^−3^/M^−1^ cm^−1^)	PMMA^b^, *λ*/nm	Solution^a^, *λ*/nm	*Φ*_PL_ b	*τ*/μs^b^	*k*_r_ × 10^−3^/s^−1^	*k*_nr_ × 10^−3^/s^−1^	(CIE*x*, CIE*y*)^b^
1	249 (44), 308 (25)	427, 454, 466	427, 451, 469	0.014	15	0.9	66	(0.16, 0.14)
[1-Cu]PF_6_	307^sh^ (38)	436, 669	—	<0.01	—	—	—	(0.52, 0.34)
[1-Ag]BF_4_	252 (16), 265 (10), 268 (11), 309 (27)	437, 482	432, 453, 470	<0.01	34	—	—	(0.18, 0.34)
[1-Ag]PF_6_	250 (18), 264 (11), 308 (25)	433, 460	434, 457, 474	0.048	16	3.0	60	(0.18, 0.20)
[1-Ag]SbF_6_	252 (15), 265 (10), 285 (10), 309 (25)	435, 470	432, 456, 474	0.016	12	1.3	82	(0.18, 0.23)
[1-Ag]BAr^F^_4_	253 (20), 267 (12), 282 (11), 311 (24)	434, 458, 475	434, 456, 476	0.14	19	7.4	45	(0.16, 0.16)
2	249 (29), 309 (23)	428, 452, 468^sh^	—	0.024	17	1.4	57	(0.16, 0.14)
[2-Ag]BAr^F^_4_	253 (21), 267 (14), 285 (13), 312 (26)	434, 459, 474	434, 459, 477	0.12	18	6.7	49	(0.16, 0.16)

a Recorded in CH_2_Cl_2_ (complexes 1, 2, [1-Ag]BAr^F^_4_, and [2-Ag]BAr^F^_4_) or CH_3_CN (complexes [1-Ag]BF_4_, [1-Ag]PF_6_, and [1-Ag]SbF_6_).

b Recorded in PMMA films at 2 wt% and at room temperature. ^sh^Shoulder.

### Cyclic voltammograms

To investigate the influence of secondary metal on the redox potentials, cyclic voltammetry was conducted on complexes 1, 2, [1-Ag]BAr^F^_4_, [1-Cu]PF_6_ and [2-Ag]BAr^F^_4_ (Fig. S10–S14†). For better comparison, the overlaid voltammogram is presented in Fig. S15† and the summary of cyclic voltammetry data is shown in Table S5.† In complexes 1 and 2, the reduction potentials are very similar and consistent with our previous works,^42,43^ although a clear oxidation wave was not located within the electrochemical window of MeCN. Upon incorporating Ag and Cu, the features attributed to the platinum acetylide complexes are retained, with reduction potentials that shift very little. In the coinage metal complexes the oxidation potentials are discernible and occur beyond 1 V *vs.* ferrocenium/ferrocene. In between the waves associated with the platinum acetylide complexes, new waves at −0.54 V (Ag^+^ complexes) and −1.02 V ([1-Cu]PF_6_) are observed, which we ascribe to the oxidation of the coinage metal ion.

## Conclusions

In this study, we introduced a new strategy to increase the photoluminescence quantum yields and radiative rate constants of blue-phosphorescent platinum acetylide complexes. This strategy, termed here as the “secondary heavy-atom effect,” involves chelation of coinage metals to *cis*-oriented pyridyl-substituted acetylide ligands. Solid-state structures and spectroscopic measurements show that aggregation can occur after introducing Cu(i) or Ag(i) to the platinum complexes. Whereas copper(i) binding shifts the luminescence to the red region, the introduction of silver(i) salts gives two layers of control over the photoluminescence. The size of the counterion controls the extent of aggregation, which influences the photoluminescence profile. With small counterions aggregation is significant in PMMA films, which results in a new red-shifted PL band that shifts the color profile out of the blue region, with aggregation-caused quenching severely reducing *Φ*_PL_. However, when the large BAr^F^_4_^−^ anion is used, aggregation is suppressed, and the blue phosphorescence profile is preserved. More significantly, in these analogues the secondary heavy-atom effect brought on by Ag(i) binding significantly improves the photoluminescence properties, substantially increasing *k*_r_ (4–8×) and *Φ*_PL_ (5–10×). This work presents a strategy to improve the properties of blue-phosphorescent platinum complexes, addressing a significant challenge in optoelectronic materials.

## Data availability

The data supporting this article have been included as part of the ESI.† Crystallographic data for 1, 2, **[**1-Cu]PF_6_, and **[**1-Ag]PF_6_ has been deposited at the CCDC under accession numbers 2394176–2394179 and can be obtained from https://www.ccdc.cam.ac.uk/structures/.

## Author contributions

Vinh Q. Dang: formal analysis, investigation, validation, visualization, writing – original draft, writing – review & editing. Chenggang Jiang: formal analysis, investigation, writing – review & editing. Thomas S. Teets: conceptualization, funding acquisition, project administration, visualization, writing – review & editing.

## Conflicts of interest

The authors declare no competing interests.

## Supplementary Material

SC-016-D5SC00172B-s001

SC-016-D5SC00172B-s002

## References

[cit1] Yersin H. , Highly Efficient Oleds With Phosphorescent Materials, Wiley-VCH, Weinheim, 2008

[cit2] Evans R. C., Douglas P., Winscom C. J. (2006). Coord. Chem. Rev..

[cit3] Jayabharathi J., Thanikachalam V., Thilagavathy S. (2023). Coord. Chem. Rev..

[cit4] Tao Y., Yang C., Qin J. (2011). Chem. Soc. Rev..

[cit5] Tankelevičiūtė E., Samuel I. D. W., Zysman-Colman E. (2024). J. Phys. Chem. Lett..

[cit6] Maganti T., Venkatesan K. (2022). ChemPlusChem.

[cit7] Bullock J. D., Valandro S. R., Sulicz A. N., Zeman C. J., Abboud K. A., Schanze K. S. (2019). J. Phys. Chem. A.

[cit8] Iridium(iii) in Optoelectronic and Photonics Applications, ed. E. Zysman-Colman, John Wiley & Sons, Inc, Chichester, West Sussex, 2017

[cit9] Pal A. K., Krotkus S., Fontani M., Mackenzie C. F. R., Cordes D. B., Slawin A. M. Z., Samuel I. D. W., Zysman-Colman E. (2018). Adv. Mater..

[cit10] Lee J., Chen H.-F., Batagoda T., Coburn C., Djurovich P. I., Thompson M. E., Forrest S. R. (2016). Nat. Mater..

[cit11] Na H., Teets T. S. (2018). J. Am. Chem. Soc..

[cit12] Cañada L. M., Kölling J., Teets T. S. (2020). Polyhedron.

[cit13] Na H., Cañada L. M., Wen Z., I-Chia Wu J., Teets T. S. (2019). Chem. Sci..

[cit14] Wu C., Shi K., Li S., Yan J., Feng Z.-Q., Tong K.-N., Zhang S.-W., Zhang Y., Zhang D., Liao L.-S., Chi Y., Wei G., Kang F. (2024). EnergyChem.

[cit15] Bin Mohd Yusoff A. R., Huckaba A. J., Nazeeruddin M. K. (2017). Top. Curr. Chem..

[cit16] Lo S.-C., Shipley C. P., Bera R. N., Harding R. E., Cowley A. R., Burn P. L., Samuel I. D. W. (2006). Chem. Mater..

[cit17] Sajoto T., Djurovich P. I., Tamayo A. B., Oxgaard J., Goddard W. A., Thompson M. E. (2009). J. Am. Chem. Soc..

[cit18] Cebrián C., Mauro M. (2018). Beilstein J. Org. Chem..

[cit19] Yersin H., Rausch A. F., Czerwieniec R., Hofbeck T., Fischer T. (2011). Coord. Chem. Rev..

[cit20] Yoon S., Teets T. S. (2024). J. Am. Chem. Soc..

[cit21] Hua F., Kinayyigit S., Cable J. R., Castellano F. N. (2005). Inorg. Chem..

[cit22] Fitzgerald S. A., Xiao X., Zhao J., Horton P. N., Coles S. J., Knighton R. C., Ward B. D., Pope S. J. A. (2023). Chem.–Eur.
J..

[cit23] Nisic F., Colombo A., Dragonetti C., Roberto D., Valore A., Malicka J. M., Cocchi M., Freeman G. R., Williams J. A. G. (2014). J. Mater. Chem. C.

[cit24] Strassner T. (2016). Acc. Chem. Res..

[cit25] Unger Y., Meyer D., Molt O., Schildknecht C., Münster I., Wagenblast G., Strassner T. (2010). Angew. Chem., Int. Ed..

[cit26] Soellner J., Pinter P., Stipurin S., Strassner T. (2021). Angew. Chem., Int. Ed..

[cit27] Zhang Y., Garg J. A., Michelin C., Fox T., Blacque O., Venkatesan K. (2011). Inorg. Chem..

[cit28] Nguyen Y. H., Wu Y., Dang V. Q., Jiang C., Teets T. S. (2024). J. Am. Chem. Soc..

[cit29] Ryu C. H., Jo U., Shin I., Kim M., Cheong K., Bin J., Lee J. Y., Lee K. M. (2024). Adv. Opt. Mater..

[cit30] She Y., Xu K., Fang X., Yang Y.-F., Lou W., Hu Y., Zhang Q., Li G. (2021). Inorg. Chem..

[cit31] Jung Y. H., Lee G. S., Muruganantham S., Kim H. R., Oh J. H., Ham J. H., Yadav S. B., Lee J. H., Chae M. Y., Kim Y.-H., Kwon J. H. (2024). Nat. Commun..

[cit32] Li G., Zhao X., Fleetham T., Chen Q., Zhan F., Zheng J., Yang Y.-F., Lou W., Yang Y., Fang K., Shao Z., Zhang Q., She Y. (2020). Chem. Mater..

[cit33] Kim J.-M., Cheong K., Jiang J., Jeon S. O., Hong W. P., Lee J. Y. (2023). Trends Chem..

[cit34] Huh J.-S., Lee D. Y., Park K. H., Kwon S.-K., Kim Y.-H., Kim J.-J. (2022). Chem. Eng. J..

[cit35] Bullock J. D., Salehi A., Zeman C. J., Abboud K. A., So F., Schanze K. S. (2017). ACS Appl. Mater. Interfaces.

[cit36] He R., Xu Z., Valandro S., Arman H. D., Xue J., Schanze K. S. (2021). ACS Appl. Mater. Interfaces.

[cit37] Zhang Y., Blacque O., Venkatesan K. (2013). Chem.–Eur. J..

[cit38] Bullock J. D., Xu Z., Valandro S., Younus M., Xue J., Schanze K. S. (2020). ACS Appl. Electron. Mater..

[cit39] Nguyen Y. H., Dang L. T. M., Cornu M., Jiang C., Darrow A. C., Dang V. Q., Teets T. S. (2024). ACS Appl. Opt. Mater..

[cit40] López-López J. C., Nguyen Y. H., Jiang C., Teets T. S. (2023). Inorg. Chem..

[cit41] Nguyen Y. H., Dang V. Q., Soares J. V., Wu J. I., Teets T. S. (2023). Chem. Sci..

[cit42] Nguyen Y. H., Soares J. V., Nguyen S. H., Wu Y., Wu J. I., Teets T. S. (2022). Inorg. Chem..

[cit43] Wu Y., Wen Z., Wu J. I., Teets T. S. (2020). Chem.–Eur. J..

[cit44] Chang C., Cheng Y., Chi Y., Chiu Y., Lin C., Lee G., Chou P., Chen C., Chang C., Wu C. (2008). Angew. Chem., Int. Ed..

[cit45] Puttock E. V., Walden M. T., Williams J. A. G. (2018). Coord. Chem. Rev..

[cit46] Bonfiglio A., Hsiao P.-W., Chen Y., Gourlaouen C., Marchand Q., César V., Bellemin-Laponnaz S., Wang Y.-X., Lu C.-W., Daniel C., Polo F., Su H.-C., Mauro M. (2022). Chem. Mater..

[cit47] Lanoë P.-H., Tong C. M., Harrington R. W., Probert M. R., Clegg W., Williams J. A. G., Kozhevnikov V. N. (2014). Chem. Commun..

[cit48] Daniels R. E., Culham S., Hunter M., Durrant M. C., Probert M. R., Clegg W., Williams J. A. G., Kozhevnikov V. N. (2016). Dalton Trans..

[cit49] Hao Z., Meng F., Wang P., Wang Y., Tan H., Pei Y., Su S., Liu Y. (2017). Dalton Trans..

[cit50] Culham S., Lanoë P.-H., Whittle V. L., Durrant M. C., Williams J. A. G., Kozhevnikov V. N. (2013). Inorg. Chem..

[cit51] Hao Z., Zhang K., Chen K., Wang P., Lu Z., Zhu W., Liu Y. (2020). Dalton Trans..

[cit52] Sun Y., Chen C., Liu B., Guo Y., Feng Z., Zhou G., Chen Z., Yang X. (2021). J. Mater. Chem. C.

[cit53] Mauro M. (2021). Chem. Commun..

[cit54] Kozhevnikov V. N., Durrant M. C., Williams J. A. G. (2011). Inorg. Chem..

[cit55] Khan A. A. T., Gobeze H. B., Islam T., Arman H. D., Schanze K. S. (2023). Dalton Trans..

[cit56] Herberhold M., Schmalz T., Milius W., Wrackmeyer B. (2002). J. Organomet. Chem..

[cit57] Wilcox S., Sethio D., Ward J. S., Frontera A., Lindh R., Rissanen K., Erdélyi M. (2022). Chem. Commun..

[cit58] London H. C., Pritchett D. Y., Pienkos J. A., McMillen C. D., Whittemore T. J., Bready C. J., Myers A. R., Vieira N. C., Harold S., Shields G. C., Wagenknecht P. S. (2022). Inorg. Chem..

[cit59] Schmidbaur H., Schier A. (2015). Angew. Chem., Int. Ed..

[cit60] Harisomayajula N. V. S., Makovetskyi S., Tsai Y. (2019). Chem.–Eur. J..

[cit61] Catalano V. J., Malwitz M. A., Etogo A. O. (2004). Inorg. Chem..

[cit62] Bocian A., Brykczyńska D., Kubicki M., Hnatejko Z., Wałęsa-Chorab M., Gorczyński A., Patroniak V. (2019). Polyhedron.

[cit63] Artem’ev A. V., Bagryanskaya I. Yu., Doronina E. P., Tolstoy P. M., Gushchin A. L., Rakhmanova M. I., Ivanov A. Y., Suturina A. O. (2017). Dalton Trans..

