# Supplementary material for: Enhanced blue phosphorescence in platinum acetylide complexes via a secondary heavy metal and anion-controlled aggregation
Source: Chem Sci. 2025 Mar 25;16(17):7302–10. doi: 10.1039/d5sc00172b (PMC11934150; doi:10.1039/d5sc00172b)
Supplement: SC-016-D5SC00172B-s001 [file SC-016-D5SC00172B-s001.pdf]

Supplementary Information (SI) for Chemical Science.
This journal is © The Royal Society of Chemistry 2025

Electronic Supplementary Information

Enhanced Blue Phosphorescence in Platinum Acetylide Complexes via a Secondary Heavy Metal and Anion-Controlled Aggregation

Vinh Q. Dang, Chenggang Jiang, and Thomas S. Teets*

University of Houston, Department of Chemistry 3585 Cullen Blvd., Room 112,

Houston, TX 77204-5003, USA

*Corresponding author: tteets@uh.edu

Contents

General procedures	S2–S3
Synthesis	S4–S8
X-ray crystallography summary	S9–S10
NMR experiments	S11–S14
Additional crystal structure figures	S15–16
Additional photophysical measurements	S17–S20
Cyclic voltammograms	S21–S24
FT-IR spectra	S25–S28
NMR spectra	S29–S39
References	S40

General procedures

Materials

Starting materials and reagents were purchased from commercial sources (Sigma-Aldrich, TCI Chemicals, and AmBeed) and used without further purification unless otherwise specified. All other solvents were dried by a commercial solvent purification system and stored over 3 Å molecular sieves. The precursors [Pt(COD)Cl_2] (COD = 1,5-cyclooctadiene) and $AgBAr^F_4$ (BAr^F_4 = tetrakis[3,5-bis(trifluoromethyl)phenyl]borate) were synthesized according to previous literature.[1,2]

Physical Methods

^1H, $^{13}C\{^1H\}$, $^{11}B\{^1H\}$, ^{19}F, and $^{31}P\{^1H\}$ NMR spectra were recorded at room temperature using a JEOL ECA-400, JEOL ECA-500, or ECA-600 NMR spectrometer. Infrared (IR) spectra were obtained on neat powders using a Thermo Nicolet Avatar FT-IR spectrometer with a diamond ATR. UV–vis absorption spectra were recorded in dichloromethane or acetonitrile in screw-capped 1 cm quartz cuvettes using an Agilent Cary 8454 UV–vis spectrophotometer. Steady-state photoluminescence (PL) and excitation spectra were recorded using a Horiba FluoroMax-4 spectrofluorometer. Air-free samples for PL spectra were prepared in a nitrogen-filled glovebox using dry, deoxygenated solvents and housed in 1 cm quartz cuvettes with septum-sealed screw caps. PL quantum yields in solution were measured with respect to a standard of quinine sulfate in 0.05 M sulfuric acid, which has a reported quantum yield (Φ_F) of 0.52.[3] The quantum yields of complexes doped into poly(methyl methacrylate) (PMMA) thin films were recorded using a Spectralon-coated integrating sphere (150 mm diameter, Labsphere) exciting at 310 nm. Phosphorescence lifetimes were measured on a Horiba DeltaFlex Lifetime System, using 330 nm excitation. Cyclic voltammograms were recorded using a CH Instruments 602E potentiostat interfaced with a nitrogen-filled glovebox. Samples were dissolved in MeCN with 0.1 M tetrabutylammonium hexafluorophosphate as the supporting electrolyte, and recorded using a glassy carbon working electrode, platinum wire counter electrode, and silver wire pseudoreference electrode. Ferrocene was added at the end of each measurement as an internal standard, and all potentials are referenced to the ferrocenium/ferrocene redox couple.

PMMA Film Fabrication

A solution of PMMA in dichloromethane or acetonitrile was prepared at room temperature inside a nitrogen-filled glovebox. Then, the respective platinum complex was added to the solution and stirred until the solution became clear. The mass concentration was set at 100 mg/mL for 1−5 wt% PMMA films and 50 mg/mL for 10 wt% PMMA films. The solution was then drop-coated onto a quartz substrate and dried at room temperature overnight prior to use.

X-ray Crystallography Details

Single crystals were mounted on a Bruker Apex II three-circle diffractometer using Mo Kα radiation (λ = 0.71073 Å). The data was collected at 123(2) K, then processed and refined within the APEXII software. Structures were solved by intrinsic phasing methods in SHELXT and refined by standard difference Fourier techniques in the program SHELX. All non-hydrogen atoms were

refined with anisotropic displacement parameters. Hydrogen atoms bonded to carbon were placed in calculated positions using the standard riding model and refined isotropically. Crystallographic details are summarized in Tables S1−S2.

Synthesis

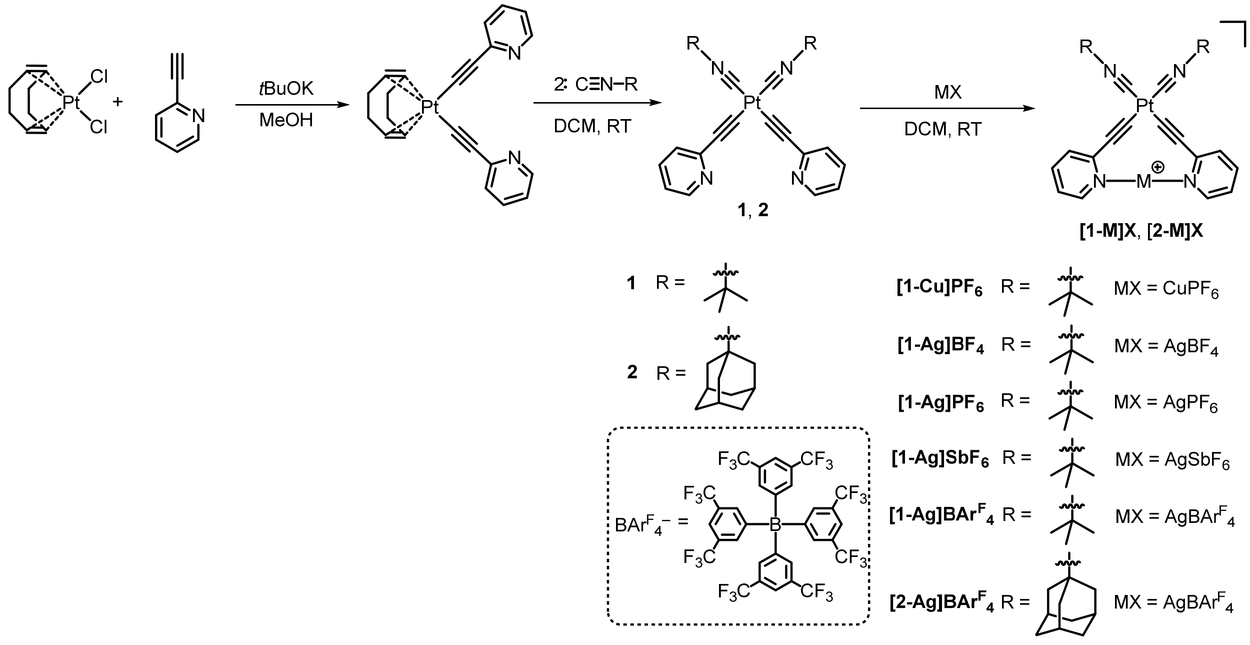

Scheme S1. Synthetic routes for new platinum complexes.

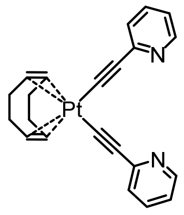

Preparation of [Pt(COD)(C≡C–2-py)$_2$]. In the glovebox, 500 mg of [Pt(COD)Cl$_2$] (1.3 mmol) was dissolved in 10 mL of methanol. In a separate vial, 380 mg of *t*BuOK (3.4 mmol) was dissolved in 10 mL of methanol, followed by the addition of 330 mg of 2-ethynylpyridine (3.2 mmol). Both vials were placed in the freezer at –37 °C for 10 minutes. Then, the solution of 2-enthynylpyridine and *t*BuOK was slowly added to the [Pt(COD)Cl$_2$] solution. The reaction mixture was stirred at room temperature for 2 h. Finally, the solid was filtered and washed with MeOH and Et$_2$O. Yield: 570 mg (84%). ^{1}H NMR (400 MHz, CHLOROFORM-*D*) δ (ppm) 8.48 (d, *J* = 4.9 Hz, 2H, Ar*H*), 7.56 (td, *J* = 7.7, 1.8 Hz, 2H, Ar*H*), 7.40 (d, *J* = 7.9 Hz, 2H, Ar*H*), 7.08 (ddd, *J* = 7.6, 4.9, 1.2 Hz, 2H, Ar*H*), 5.77 (s with ^{195}Pt satellites, $^3J_{Pt-H}$ = 44, 4H, COD), 2.56 (s, 8H, COD). These data matched what is reported in the literature.[4]

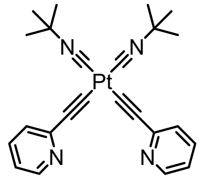

1

Preparation of *cis*-[Pt(CN–*t*Bu)$_2$(C≡C–2-py)$_2$] (1). In the glovebox, 200 mg of [Pt(COD)(C≡C–2-py)$_2$] (0.40 mmol) was dissolved in 20 mL of CH_2Cl_2. Then, *tert*-butyl isocyanide (79 mg, 0.95 mmol) was added to Pt(COD)(C≡C–2-py)$_2$ solution. The mixture was stirred at room temperature for 1 h. The solvent was removed under vacuum. The product was purified via precipitation from CH_2Cl_2/Et_2O, collected by filtration, and washed with Et_2O. Yield: 178 mg (79%). ^{1}H NMR (400 MHz, ACETONITRILE-*D*$_3$) δ (ppm) 8.39 (ddd, *J* = 4.9, 1.9, 1.0 Hz, 2H, Ar*H*), 7.57 (td, *J* = 7.7, 1.9 Hz, 2H, Ar*H*), 7.23 (dt, *J* = 7.9, 1.1 Hz, 2H, Ar*H*), 7.08 (ddd, *J* = 7.6, 4.9, 1.2 Hz, 2H, Ar*H*), 1.54 (s, 18H, –C*H*$_3$). ^{13}C{^{1}H} NMR (151 MHz, ACETONITRILE-*D*$_3$) δ (ppm) 149.5 (Ar), 146.1 (Ar), 135.9 (Ar), 126.6 (Ar), 122.1 (t, $^1J^{13}C≡^{14}N$ = 22 Hz, *C*≡N–*t*Bu), 121.0 (Ar), 105.9 (s, ^{195}Pt satellites, $^2J_{Pt-C}$ = 330 Hz, Pt–C≡*C*–py), 95.1 (s, ^{195}Pt satellites, $^1J_{Pt-C}$ = 1178 Hz, Pt–*C*≡C–py), 59.2 [–*C*(CH$_3$)$_3$], 29.1 (–*C*H$_3$). FT-IR (cm^{-1}): 2232 ($\tilde{\nu}_{CN}$), 2211 ($\tilde{\nu}_{CN}$), 2130 ($\tilde{\nu}_{CC}$).

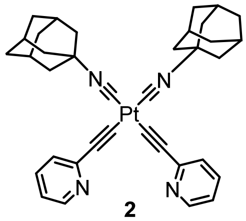

2

Preparation of *cis*-[Pt(CN–*adamantyl*)$_2$(C≡C–2-py)$_2$] (2). In the glovebox, 200 mg of [Pt(COD)(C≡C–2-py)$_2$] (0.40 mmol) was dissolved in 20 mL of CH_2Cl_2. Then, 1-adamantyl isocyanide (153 mg, 0.95 mmol) was added to the Pt(COD)(C≡C–2-py)$_2$ solution. The mixture was stirred at room temperature for 1 h. The solvent was removed under vacuum, and the product was purified via precipitation from CH_2Cl_2/pentane, collected by filtration, and washed with pentane. Yield: 213 mg (74%). ^{1}H NMR (400 MHz, CHLOROFORM-*D*) δ (ppm) 8.43 (d, *J* = 3.8 Hz, 2H, Ar*H*), 7.48 (td, *J* = 7.7, 1.9 Hz, 2H, Ar*H*), 7.39 (d, *J* = 7.9 Hz, 2H, Ar*H*), 6.99 (ddd, *J* = 7.5, 4.9, 1.3 Hz, 2H, Ar*H*), 2.12 (s, 14H, −adamantly group), 1.90 (s, 4H, −adamantly group), 1.71 – 1.63 (m, 12H, −adamantly group). ^{13}C{^{1}H} NMR (151 MHz, CHLOROFORM-*D*) δ (ppm) 149.1 (Ar), 146.5 (Ar), 135.4 (Ar), 127.3 (Ar), 123.6 (*C*≡N–adamantyl), 120.6 (Ar), 105.8 (Pt–C≡*C*–py), 95.1 (Pt–*C*≡C–py), 58.3 (−adamantly group), 42.7 (−adamantly group), 35.3 (−adamantly group), 28.8 (−adamantly group). FT-IR (cm^{-1}): 2228 ($\tilde{\nu}_{CN}$), 2206 ($\tilde{\nu}_{CN}$), 2131 ($\tilde{\nu}_{CC}$).

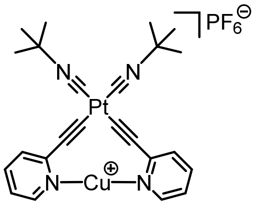

[1-Cu]PF$_6$

Preparation of complex [1-Cu]PF$_6$. In the glovebox, 50 mg of complex **1** (0.088 mmol) was dissolved in 10 mL of CH_2Cl_2. A solution of $[Cu(CH_3CN)_4]PF_6$ (41 mg, 0.13 mmol) in 5 mL of CH_2Cl_2 was added to the solution of complex **1**. The mixture was stirred overnight at RT. The solid was filtered, washed with CH_2Cl_2, and dried under vacuum. Yield: 52 mg (76%). ^{1}H NMR (500 MHz, ACETONITRILE-D_3) δ (ppm) 8.63 (d, J = 5.4 Hz, 2H, Ar*H*), 7.76 (t, J = 7.8 Hz, 2H, Ar*H*), 7.36 – 7.32 (m, 4H, Ar*H*), 1.50 (s, 18H, –C*H*$_3$). ^{19}F NMR (470 MHz, ACETONITRILE-D_3) δ (ppm) −72.8 (d, J = 707 Hz, 6F, PF$_6$). ^{31}P{^{1}H} NMR (202 MHz, ACETONITRILE-D_3) δ (ppm) −144.0 (sept, J = 707 Hz, 1P, PF$_6$). FT-IR (cm^{-1}): 2225 ($\tilde{\nu}_{CN}$), 2135 ($\tilde{\nu}_{CC}$).

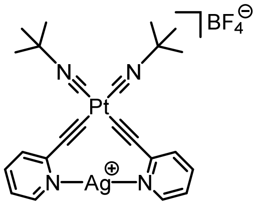

[1-Ag]BF$_4$

Preparation of complex [1-Ag]BF$_4$. In the glovebox, 50 mg of complex **1** (0.088 mmol) was dissolved in 10 mL of CH_2Cl_2. A solution of $AgBF_4$ (26 mg, 0.13 mmol) or $[Ag(CH_3CN)_4]BF_4$ (47 mg, 0.13 mmol) in 5 mL of CH_2Cl_2 was added to the solution of complex **1**. A solid formed after adding $AgBF_4$. The mixture was stirred for 1 h at RT. The solid was filtered and washed with CH_2Cl_2. The product was dried under vacuum. Yield: 55 mg (82%). ^{1}H NMR (400 MHz, ACETONITRILE-D_3) δ (ppm) 8.52 (d, J = 5.3 Hz, 2H, Ar*H*), 7.78 (t, J = 7.8 Hz, 2H, Ar*H*), 7.37 (t, J = 6.6 Hz, 2H, Ar*H*), 7.29 (d, J = 7.9 Hz, 2H, Ar*H*), 1.57 (s, 18H, –C*H*$_3$). ^{19}F NMR (376 MHz, ACETONITRILE-D_3) δ (ppm) −151.6. ^{11}B{^{1}H} NMR (128 MHz, ACETONITRILE-D_3) δ (ppm) −2.2. FT-IR (cm^{-1}): 2240 ($\tilde{\nu}_{CN}$), 2225 ($\tilde{\nu}_{CN}$), 2109 ($\tilde{\nu}_{CC}$).

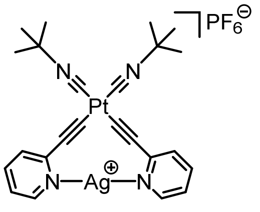

[1-Ag]PF$_6$

Preparation of complex [1-Ag]PF$_6$. In the glovebox, 50 mg of complex **1** (0.088 mmol) was dissolved in 10 mL of CH_2Cl_2. A solution of $AgPF_6$ (33 mg, 0.13 mmol) in 5 mL of CH_2Cl_2 was added to the solution of complex **1**. A solid precipitated after adding $AgPF_6$. The mixture was stirred for 1 h at RT. The solid was filtered and washed with CH_2Cl_2. The product was dried under

vacuum. Yield: 60 mg (83%). ^{1}H NMR (400 MHz, ACETONITRILE-D_3) δ (ppm) 8.52 (ddd, J = 5.3, 1.8, 1.0 Hz, 2H, Ar*H*), 7.37 (ddd, J = 7.6, 5.3, 1.3 Hz, 2H, Ar*H*), 7.32 (d, J = 7.9 Hz, 2H, Ar*H*), 1.57 (s, 18H, –C*H*$_3$). ^{19}F NMR (470 MHz, ACETONITRILE-D_3) δ (ppm) −72.8 (d, J = 707 Hz, 6F, PF_6). ^{31}P{^{1}H} NMR (202 MHz, ACETONITRILE-D_3) δ (ppm) −144.0 (sept, J = 707 Hz, 1P, PF_6). FT-IR (cm^{-1}): 2240 ($\tilde{\nu}_{CN}$), 2225 ($\tilde{\nu}_{CN}$), 2109 ($\tilde{\nu}_{CC}$).

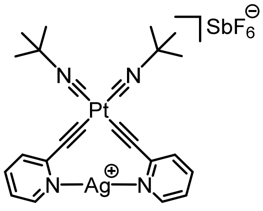

[1-Ag]SbF$_6$

Preparation of complex [1-Ag]SbF$_6$. In the glovebox, 25 mg of complex **1** (0.044 mmol) was dissolved in 5 mL of CH_2Cl_2. A solution of $AgSbF_6$ (23 mg, 0.066 mmol) in 5 mL of CH_2Cl_2 was added to the solution of complex **1**, forming a solid. The mixture was stirred for 1 h at RT. The solid was filtered and washed with CH_2Cl_2. The product was dried under vacuum. Yield: 34 mg (86%). ^{1}H NMR (500 MHz, ACETONITRILE-D_3) δ (ppm) 8.52 (ddd, J = 5.3, 1.8, 0.9 Hz, 2H, Ar*H*), 7.81 (td, J = 7.8, 1.7 Hz, 2H, Ar*H*), 7.38 (ddd, J = 7.7, 5.3, 1.3 Hz, 2H, Ar*H*), 7.35 (dt, J = 7.9, 1.1 Hz, 2H, Ar*H*), 1.57 (s, 18H, –C*H*$_3$). ^{19}F NMR (470 MHz, ACETONITRILE-D_3) δ (ppm) −196.3. FT-IR (cm^{-1}): 2256 ($\tilde{\nu}_{CN}$), 2244 ($\tilde{\nu}_{CN}$), 2070 ($\tilde{\nu}_{CC}$).

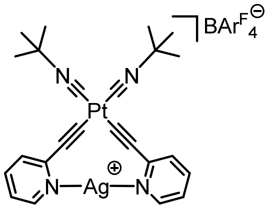

[1-Ag]BAr$^{F}_{4}$

Preparation of complex [1-Ag]BAr$^{F}_{4}$. In the glovebox, 25 mg of complex **1** (0.044 mmol) was dissolved in 5 mL of CH_2Cl_2. A solution of AgBAr$^{F}_{4}$ (50 mg, 0.052 mmol) in 5 mL of DCM was added to the solution of complex **1**. The mixture was stirred for 1 h at RT. Then, the solvent was removed under vacuum. Et_2O was added to the crude product, and the mixture was stirred for 10 min. The white solid product was collected and washed with Et_2O. Finally, the product was dried under vacuum overnight. Yield: 51 mg (75%). ^{1}H NMR (400 MHz, ACETONITRILE-D_3) δ (ppm) 8.52 (d, J = 5.2 Hz, 2H, Ar*H*), 7.67 – 7.64 (m, 12H, Ar*H*), 7.39 – 7.34 (m, 4H, Ar*H*), 1.57 (s, 18H, –C*H*$_3$). ^{19}F NMR (470 MHz, ACETONITRILE-D_3) δ (ppm) −63.1. ^{11}B{^{1}H} NMR (160 MHz, ACETONITRILE-D_3) δ (ppm) −7.7. FT-IR (cm^{-1}): 2238 ($\tilde{\nu}_{CN}$), 2219 ($\tilde{\nu}_{CN}$), 2142 ($\tilde{\nu}_{CC}$).

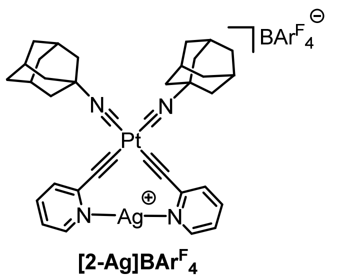

[2-Ag]BAr$^F_4$

Preparation of complex [2-Ag]BAr$^F_4$. In the glovebox, 25 mg of complex **2** (0.035 mmol) was dissolved in 5 mL of CH_2Cl_2. A solution of AgBAr$^F_4$ (41 mg, 0.042 mmol) in 5 mL of CH_2Cl_2 was added to the solution of complex **2**. The solution was stirred for 1 h at RT. Then, the solvent was removed under vacuum. The product was purified via precipitation from CH_2Cl_2/pentane. The product was collected by filtration and washed with pentane. Yield: 40 mg (67%). ^{1}H NMR (400 MHz, ACETONITRILE-D_3) δ (ppm) 8.50 (d, J = 5.0 Hz, 2H, Ar*H*), 7.77 (td, J = 7.8, 1.7 Hz, 2H, Ar*H*), 7.68 – 7.64 (m, 12H, Ar*H*), 7.35 (ddd, J = 7.7, 5.3, 1.3 Hz, 2H, Ar*H*), 7.29 (dt, J = 7.9, 1.1 Hz, 2H, Ar*H*), 2.15 – 2.13 (m, 18H, −adamantly group), 1.75 – 1.71 (m, 12H, −adamantly group). ^{19}F NMR (376 MHz, ACETONITRILE-D_3) δ (ppm) −63.1. ^{11}B{^{1}H} NMR (128 MHz, ACETONITRILE-D_3) δ (ppm) −7.7. FT-IR (cm^{-1}): 2234 ($\tilde{\nu}_{CN}$), 2218 ($\tilde{\nu}_{CN}$), 2139 ($\tilde{\nu}_{CC}$).

X-ray Crystallography Summary

Table S1. Summary of crystallographic data for complexes **1** and **2**.

	**1**	**2**
CCDC	2394176	2394177
Chemical formula	$C_{24}H_{27}N_4Pt$	$C_{36}H_{38}N_4Pt$
M_r	566.58	721.79
Crystal system, space group	Orthorhombic, $Pca2_1$	Triclinic, $P\bar{1}$
Temperature (K)	123	123
a, b, c (Å)	26.4912(17), 11.0501(7), 22.1450(15)	11.975(3), 15.698(4), 19.951(5)
α, β, γ (°)	90, 90, 90	79.386(3), 84.090(4), 71.728(3)
V (Å^3)	6482.5(7)	3496.4(16)
Z	8	4
μ (mm^{-1})	4.34	4.04
Crystal size (mm)	0.22 × 0.09 × 0.04	0.16 × 0.08 × 0.03
T_{min}, T_{max}	0.567, 0.746	0.597, 0.746
No. of measured, independent and observed [$I > 2\sigma(I)$] reflections	37826, 14783, 12168	43231, 13257, 9388
R_{int}	0.044	0.058
$(\sin \theta/\lambda)_{max}$ (Å^{-1})	0.651	0.610
$R[F^2 > 2\sigma(F^2)]$, $wR(F^2)$, S	0.033, 0.066, 1.02	0.036, 0.079, 0.98
No. of reflections	14783	13257
No. of parameters	536	821
No. of restraints	915	2178
$\Delta\rho_{max}$, $\Delta\rho_{min}$ (e Å^{-3})	1.16, −0.80	0.73, −0.95

Table S2. Summary of crystallographic data for complexes **[1-Cu]PF₆** and **[1-Ag]PF₆**.

	**[1-Cu]PF$_6$**·CH_2Cl_2·$C_4H_{10}O$	**[1-Ag]PF$_6$**·2(CH_2Cl_2)
CCDC	2394178	2394179
Chemical formula	$C_{48}H_{52}Cu_2N_8Pt_2$·2(PF_6)·CH_2Cl_2·$C_4H_{10}O$	$C_{48}H_{52}Ag_2N_8Pt_2$·2(PF_6)·2(CH_2Cl_2)
M_r	1707.22	1806.68
Crystal system, space group	Triclinic, $P\bar{1}$	Monoclinic, $P2_1/n$
Temperature (K)	123	123
a, b, c (Å)	10.1312(18), 13.882(3), 25.808(5)	11.611(5), 13.011(6), 21.235(9)
α, β, γ (°)	89.634(2), 78.885(2), 75.512(2)	90, 101.158(5), 90
V (Å^3)	3445.0(11)	3147(2)
Z	2	2
μ (mm^{-1})	4.85	5.34
Crystal size (mm)	0.28 × 0.18 × 0.05	0.21 × 0.09 × 0.08
T_{min}, T_{max}	0.520, 0.746	0.562, 0.746
No. of measured, independent and observed [$I > 2\sigma(I)$] reflections	50989, 13087, 10157	15284, 5558, 4517
R_{int}	0.062	0.051
(sin θ/λ)$_{max}$ (Å^{-1})	0.610	0.595
$R[F^2 > 2\sigma(F^2)]$, $wR(F^2)$, S	0.037, 0.091, 1.06	0.048, 0.099, 1.06
No. of reflections	13087	5558
No. of parameters	753	367
No. of restraints	700	0
$\Delta\rho_{max}$, $\Delta\rho_{min}$ (e Å^{-3})	1.25, −1.72	1.94, −1.94

Additional experiments

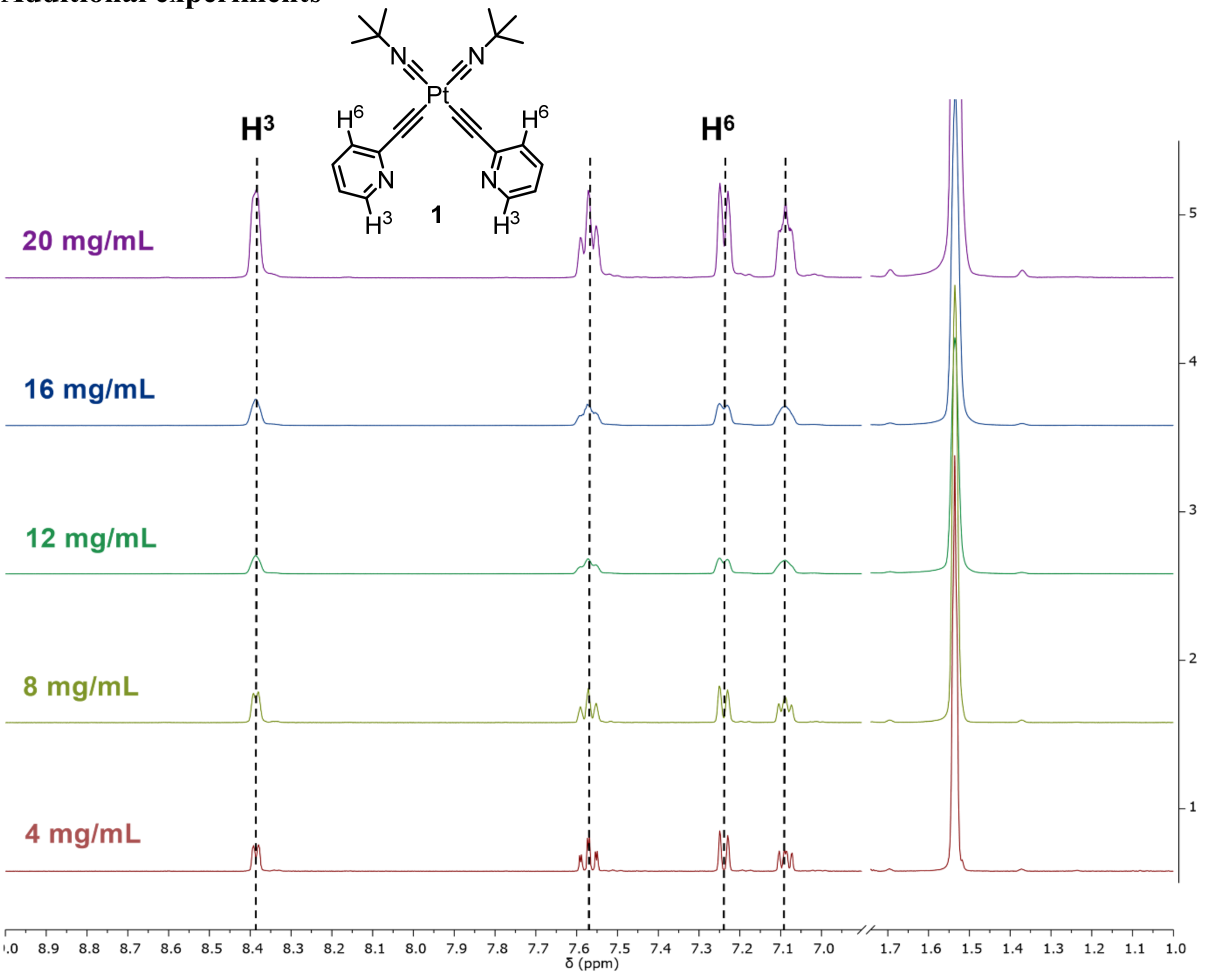

Fig. S1 ^{1}H NMR spectra of complex **1** in CD_3CN with different concentrations.

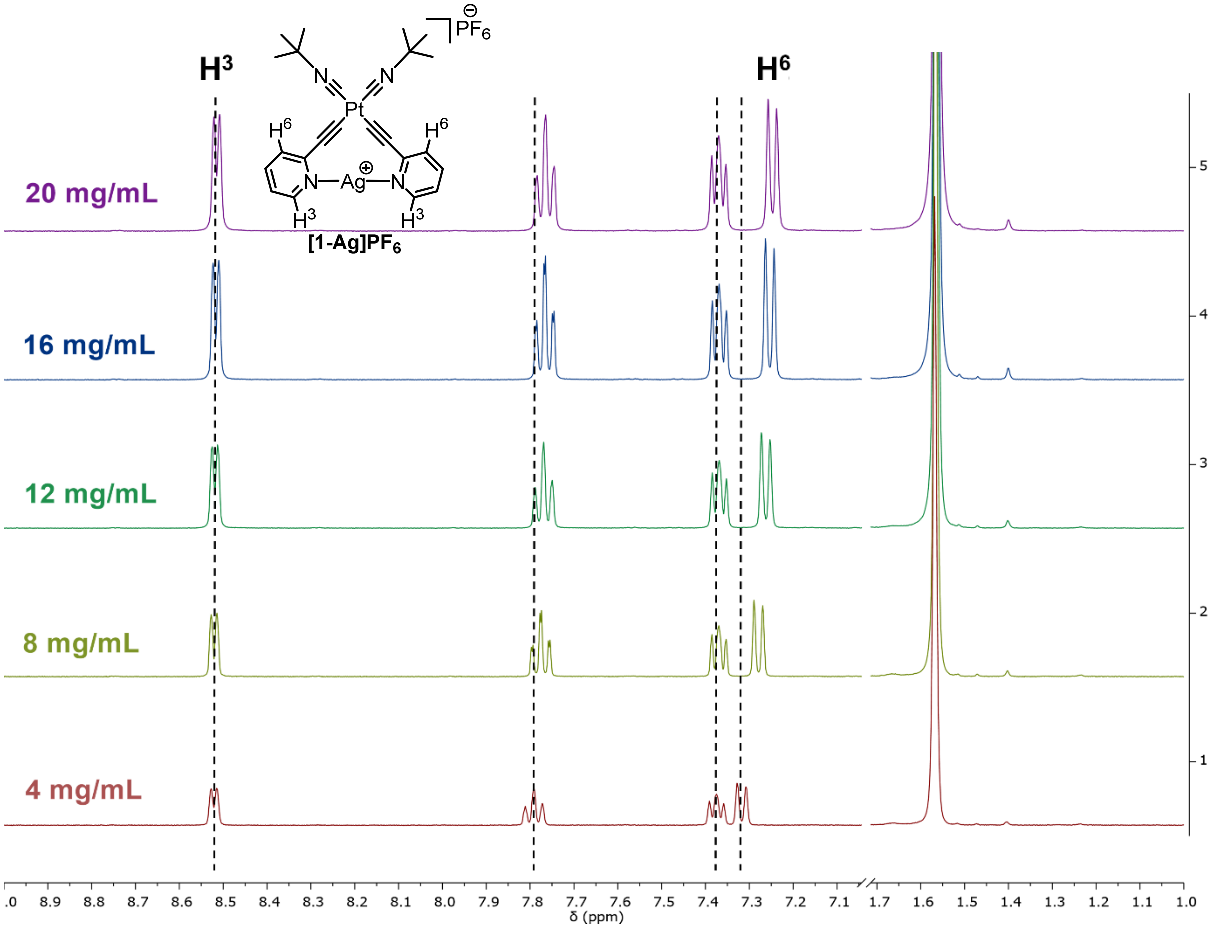

Fig. S2 ^{1}H NMR spectra of complex **[1-Ag]PF$_6$** in CD$_3$CN with different concentrations.

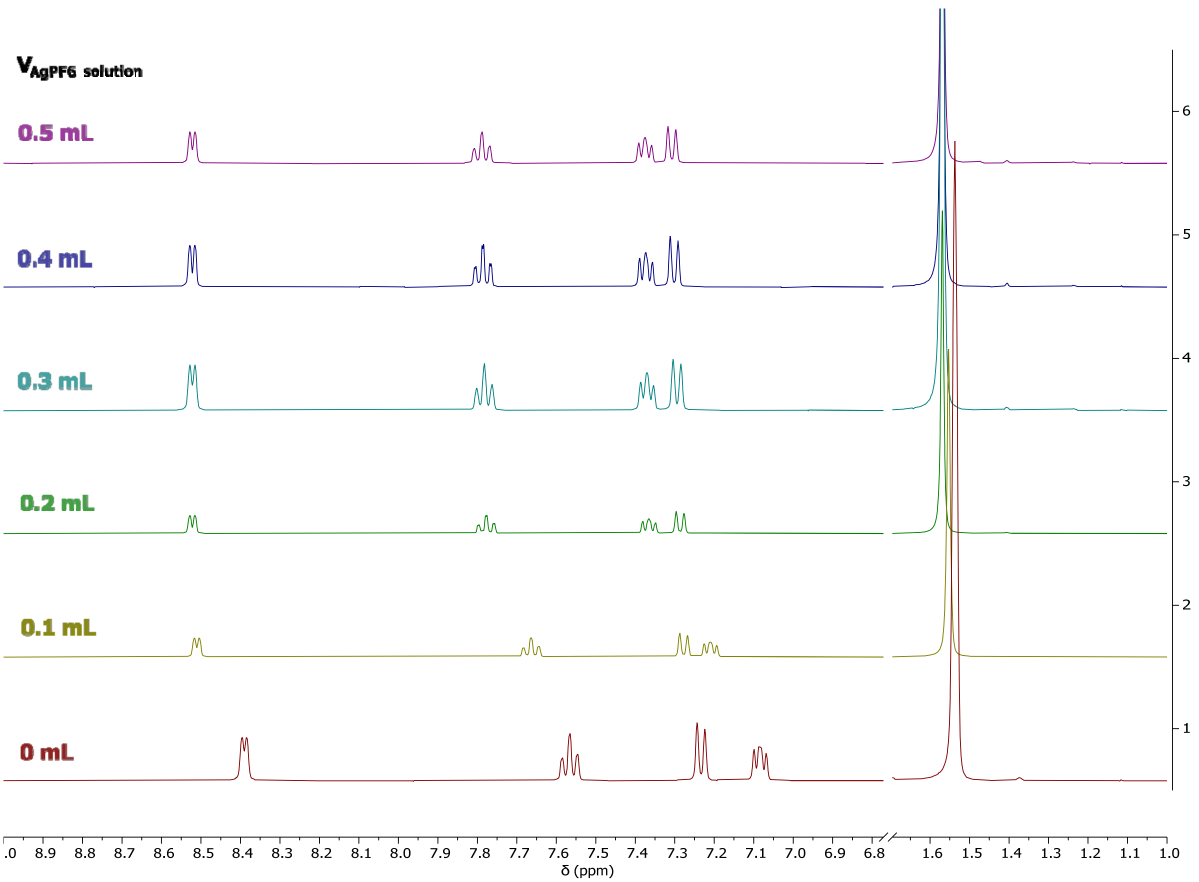

Fig. S3 ^{1}H NMR spectra of complex **1** in CD_3CN with different amounts of $AgPF_6$. The solutions of complex **1** (5 mg, 0.009 mmol in 1 mL of CD_3CN) and $AgPF_6$ (8 mg, 0.032 mmol in 1 mL of CD_3CN) were prepared. The NMR tube was filled with 1 mL of complex **1** solution. The ^{1}H NMR spectra were recorded in the absence of $AgPF_6$ and after adding different amounts of $AgPF_6$ stock solution. mol **1** = mol $AgPF_6$ when the added volume of $AgPF_6$ solution = 0.28 mL.

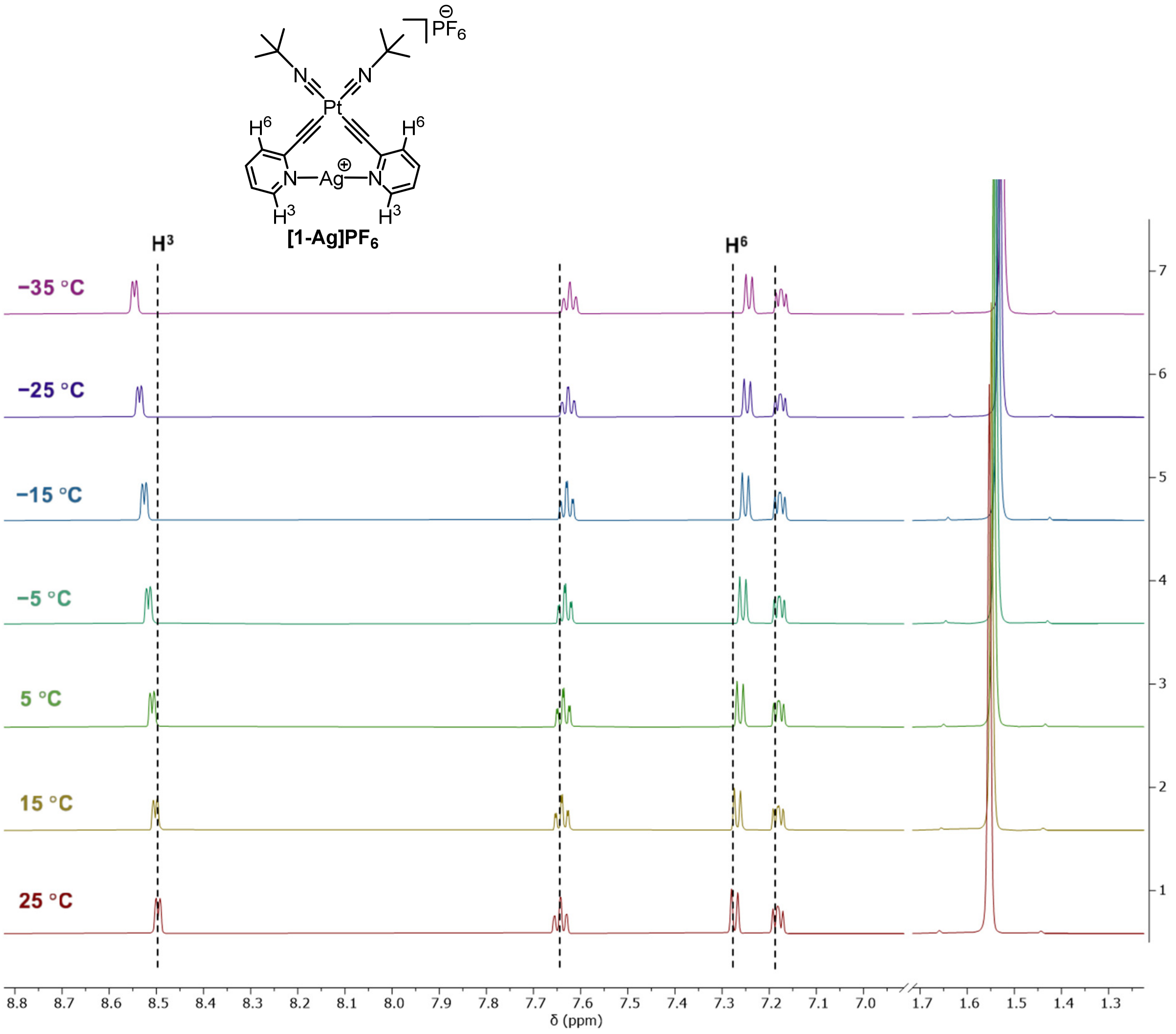

Fig. S4 ^{1}H NMR spectra of the mixture complex **1** and $AgPF_6$ in CD_3CN with different temperatures. The solutions of complex **1** (5 mg, 0.009 mmol in 1 mL of CD_3CN) and $AgPF_6$ (8 mg, 0.032 mmol in 1 mL of CD_3CN) were prepared. The NMR tube was filled with 1 mL of complex **1** solution and 0.1 mL of $AgPF_6$ solution. The ^{1}H NMR spectra were recorded over the temperature range of 25 °C to −35 °C.

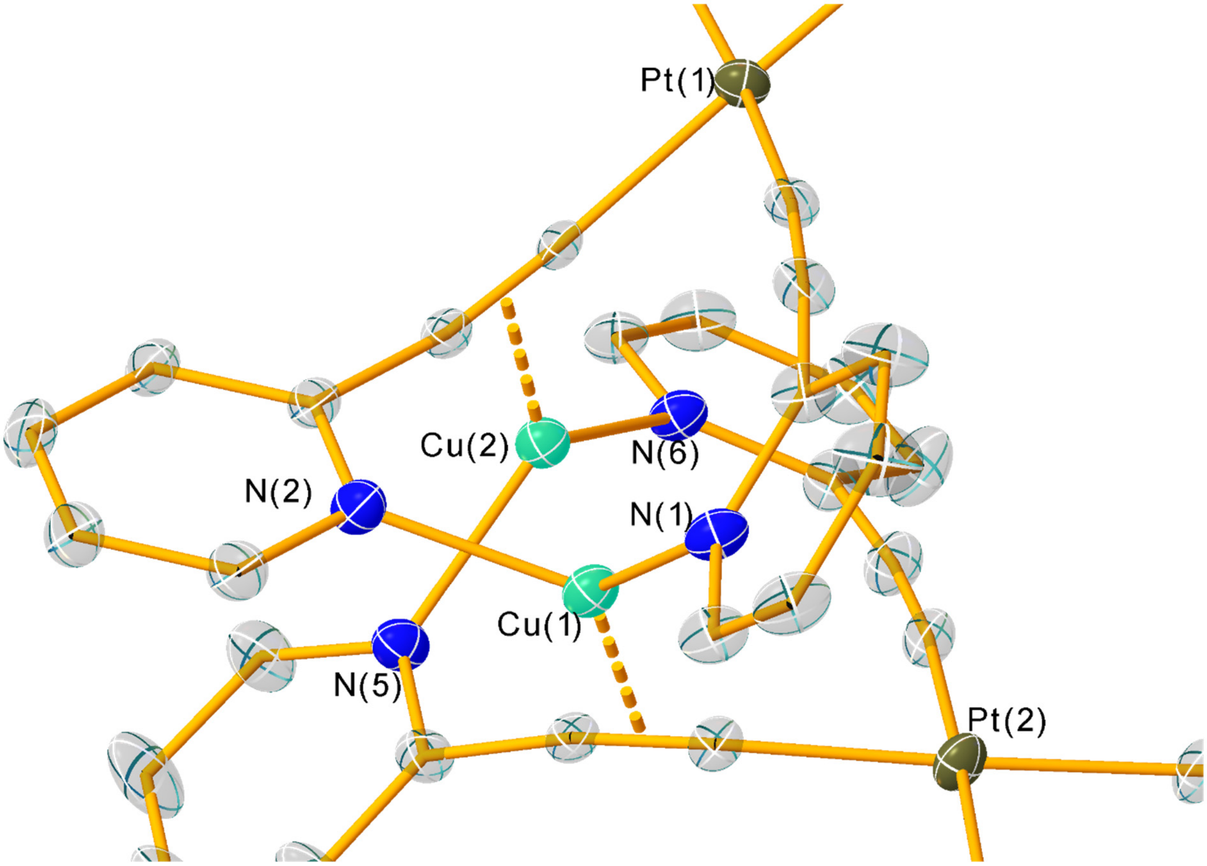

Fig. S5. Magnification of the structure of **[1-Cu]PF$_6$**, determined from single-cyrstal X-ray diffraction, zooming in on the secondary interactions involving the Cu(I) ions. Carbon atoms are displayed translucently for clarity.

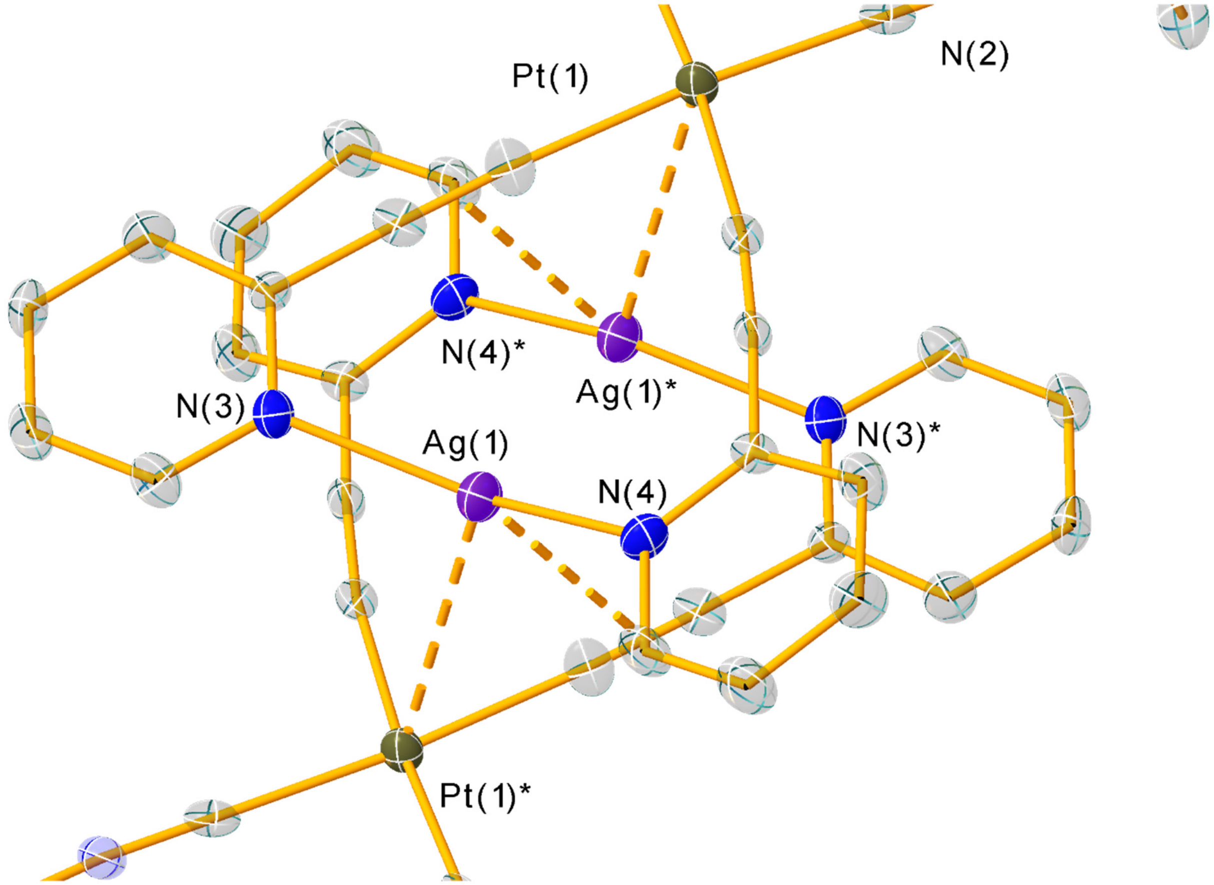

Fig. S6. Magnification of the structure of **[1-Ag]PF$_6$**, determined from single-cyrstal X-ray diffraction, zooming in on the secondary interactions involving the Ag(I) ions. Carbon atoms and a noncoordinated nitrogen atom are displayed translucently for clarity.

Photophysical Measurements

Table S3. Summary of photoluminescence data in solution[a]

Complex	Φ_{PL}	τ/μs	$k_r\times10^{-4}$/s^{-1}	$k_{nr}\times10^{-4}$/s^{-1}
**1**	0.0009	0.14	0.64	714
**[1-Cu]PF$_6$**[b]	-	-	-	-
**[1-Ag]BF$_4$**	0.003	0.51	0.59	195
**[1-Ag]PF$_6$**	0.005	0.31	1.6	321
**[1-Ag]SbF$_6$**	0.004	0.35	1.1	285
**[1-Ag]BAr$^F_4$**	0.002	0.39	0.51	256
**2**[b]	-	-	-	-
**[2-Ag]BAr$^F_4$**	0.004	0.35	1.1	284

[a] Recorded in CH_2Cl_2 (complexes **1**, **2**, **[1-Ag]BArF**, and **[2-Ag]BArF**) or CH_3CN (complexes [**1-Ag]BF$_4$**, **[1-Ag]PF$_6$**, and **[1-Ag]SbF$_6$**). [b] No photoluminescence in solution.

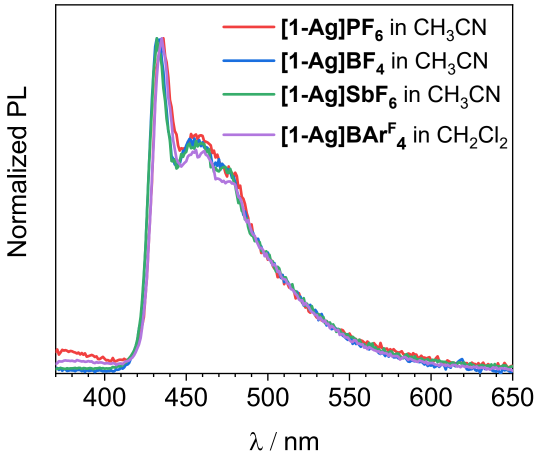

Fig. S7 Photoluminescence spectra of complexes [**1-Ag]X** in solution.

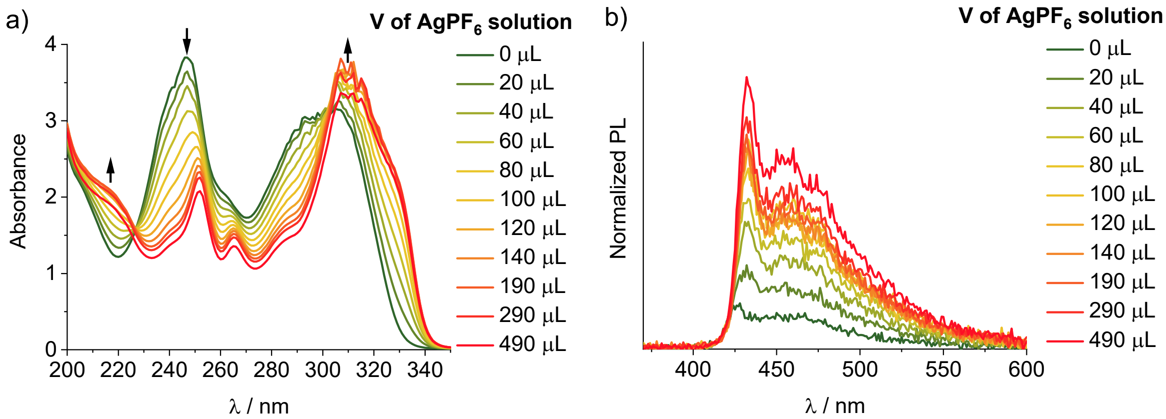

Fig. S8 UV–vis absorption (a) and photoluminescence spectra (b) of complex **1** in CH_3CN with different amounts of $AgPF_6$. The stock solutions of complex **1** (1.3 mg, 0.0023 mmol in 1 mL of CH_3CN) and $AgPF_6$ (1.2 mg, 0.0047 mmol in 1.5 mL of CH_3CN) were prepared inside the glovebox. The cuvette was filled with 3 mL of CH_3CN and 200 μL of complex **1** stock solution. The absorption and PL spectra were recorded in the absence of $AgPF_6$ and after adding different amounts of $AgPF_6$ stock solution. mol **1** = mol $AgPF_6$ when the volume of $AgPF_6$ solution = 147 μL.

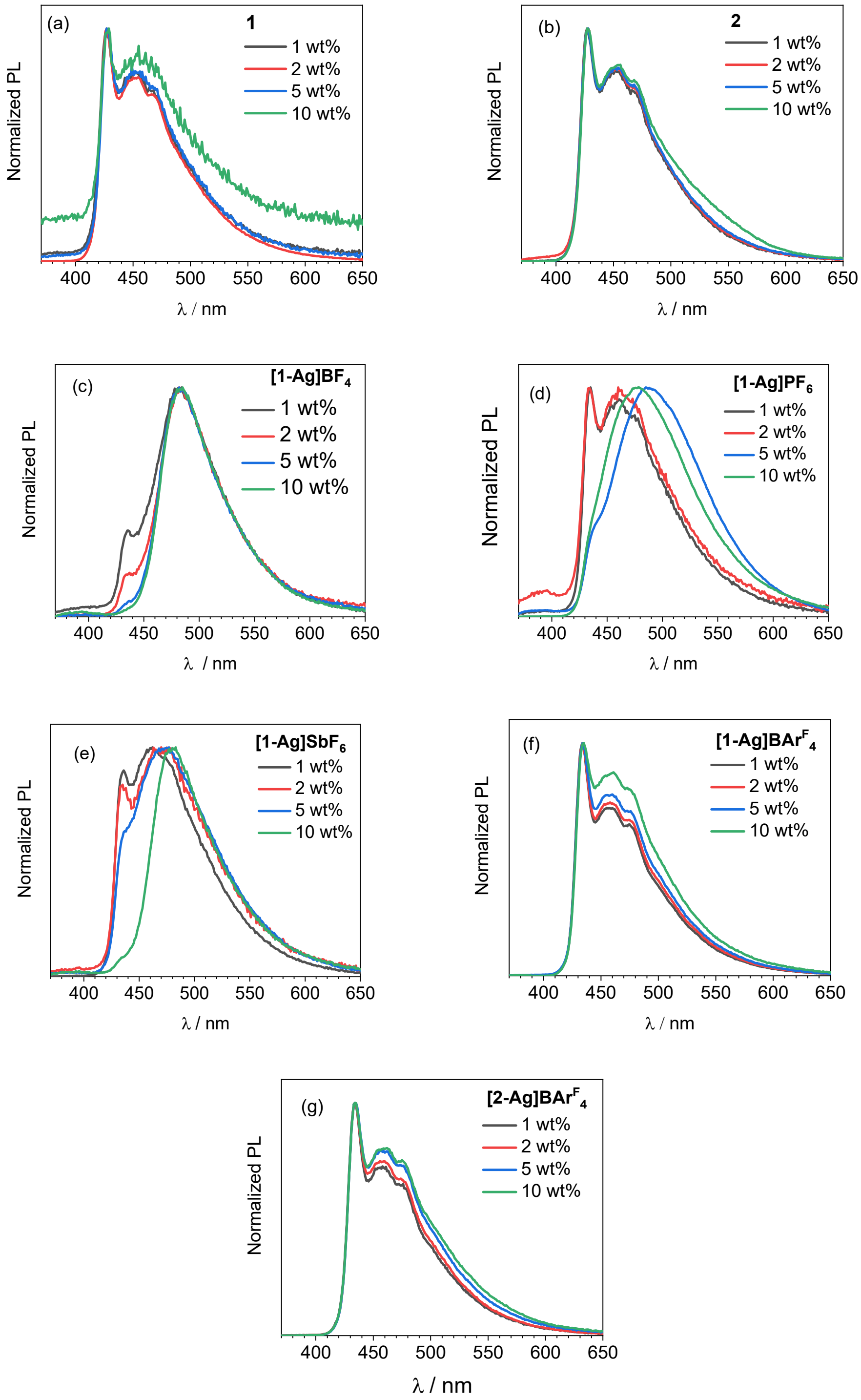

Fig. S9 PL spectra of new platinum complexes in PMMA films with different concentrations. Samples were excited at λ = 310 nm.

Table S4. Summary of photoluminescence quantum yields in PMMA films with different concentrations.

Complex	Φ_{PL}			
	1 wt%	2 wt%	5 wt%	10 wt%
**1**	0.034	0.014	0.017	0.006
**[1-Ag]BF$_4$**	0.045	< 0.01	< 0.01	< 0.01
**[1-Ag]PF$_6$**	0.060	0.048	< 0.01	< 0.01
**[1-Ag]SbF$_6$**	0.071	0.016	< 0.01	< 0.01
**[1-Ag]BAr$^F_4$**	0.16	0.14	0.05	0.03
**2**	0.032	0.024	0.024	0.017
**[2-Ag]BAr$^F_4$**	0.14	0.12	0.074	0.027

Cyclic voltammograms

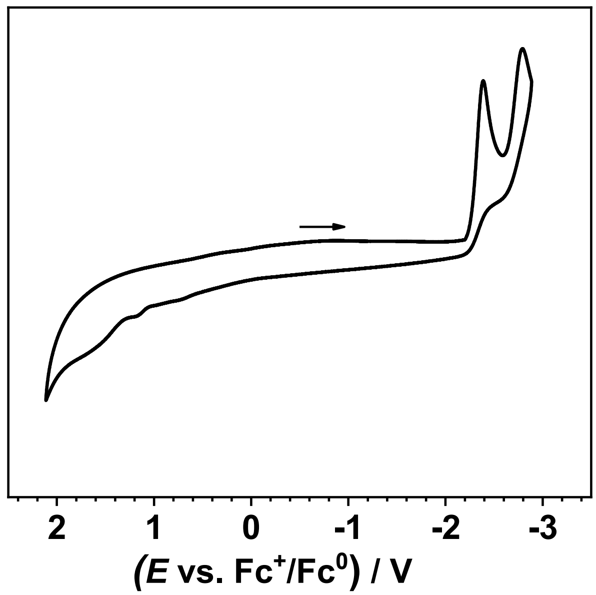

Fig. S10 Cyclic voltammogram of complex **1**, recorded in MeCN with 0.1 M $(NBu_4)(PF_6)$ supporting electrolyte. The arrow shows the scan direction.

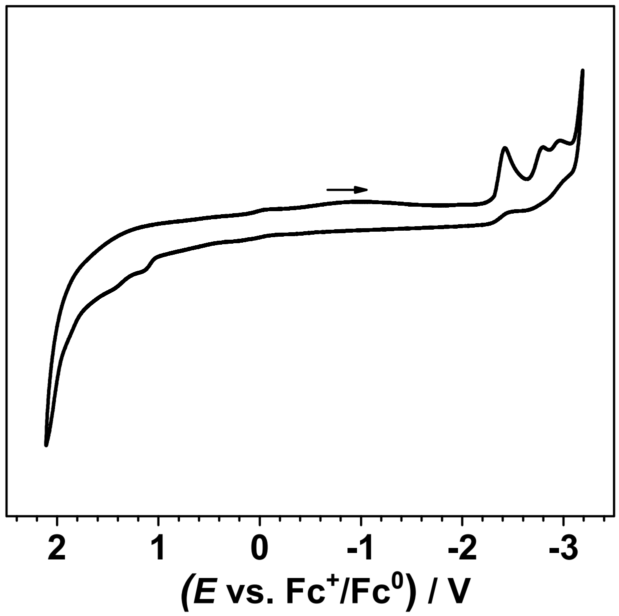

Fig. S11 Cyclic voltammogram of complex **2**, recorded in MeCN with 0.1 M $(NBu_4)(PF_6)$ supporting electrolyte. The arrow shows the scan direction.

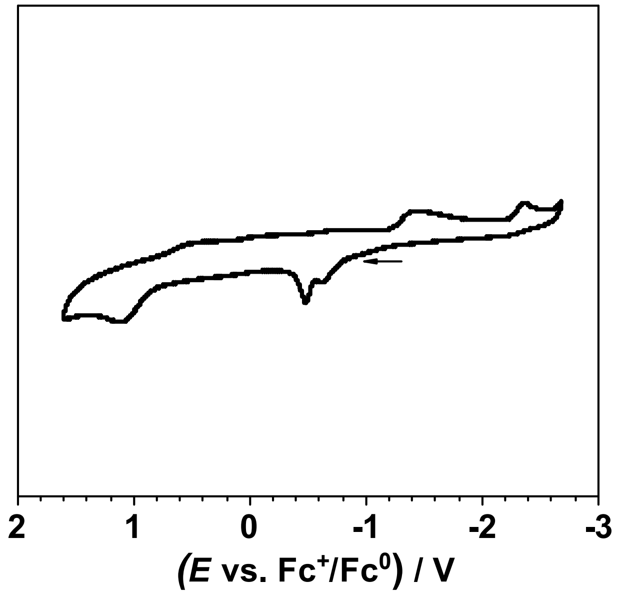

Fig. S12 Cyclic voltammogram of complex **[1-Cu]PF_6**, recorded in MeCN with 0.1 M $(NBu_4)(PF_6)$ supporting electrolyte. The arrow shows the scan direction.

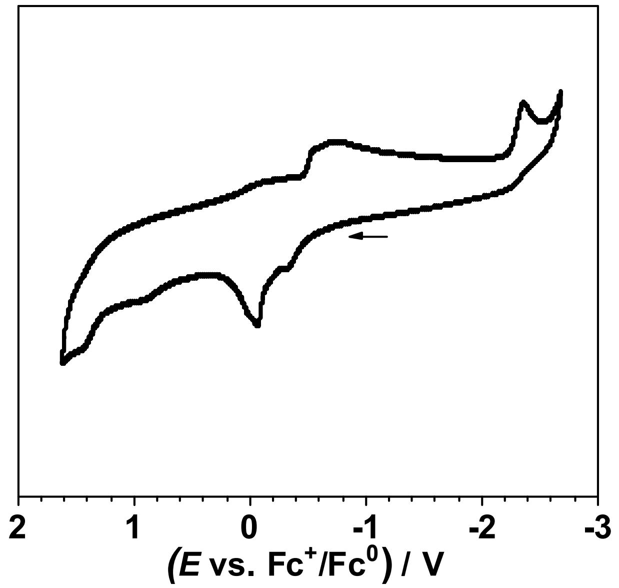

Fig. S13 Cyclic voltammogram of complex **[1-Ag]BAr^F_4**, recorded in MeCN with 0.1 M $(NBu_4)(PF_6)$ supporting electrolyte. The arrow shows the scan direction.

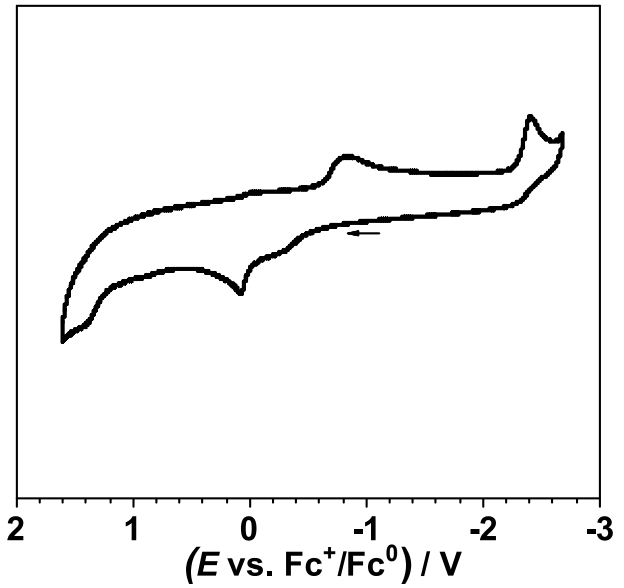

Fig. S14 Cyclic voltammogram of complex **[2-Ag]BAr^{F}$_4$**, recorded in MeCN with 0.1 M $(NBu_4)(PF_6)$ supporting electrolyte. The arrow shows the scan direction.

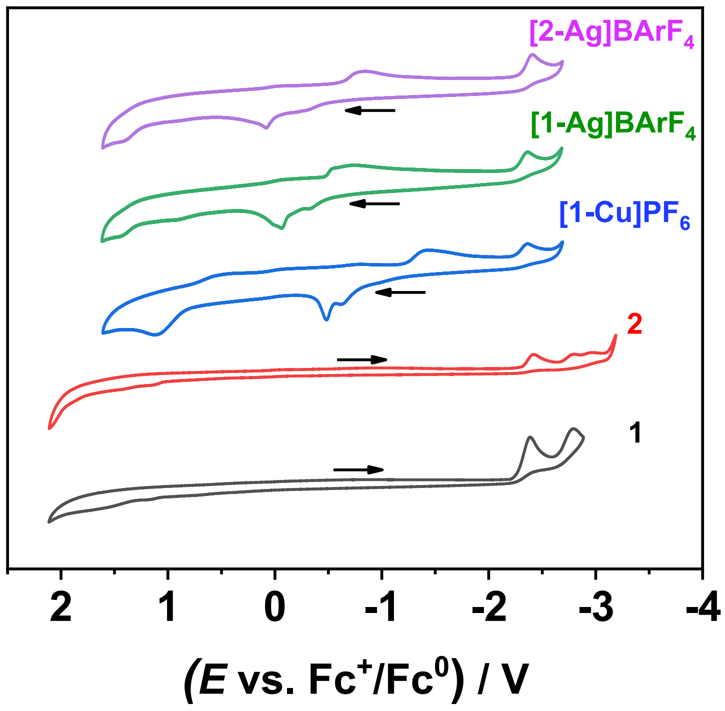

Fig. S15 The overlaid cyclic voltammograms of complexes **1**, **2**, **[1-Cu]PF$_6$**, **[1-Ag]BAr^{F}$_4$**, and **[2-Ag]BAr^{F}$_4$**.

Table S5. Summary of cyclic voltammetry data.

Complex	E^{ox} (Pt complex) (V vs Fc^+/Fc)[a]	E^{ox} (M^{2+}/M^+) (V vs Fc^+/Fc)[b]	E^{red} (Pt complex) (V vs Fc^+/Fc)[a]
**1**	N.D.[c]	-	–2.31
**2**	N.D.[c]	-	–2.36
**[1-Cu]PF$_6$**	+0.95	–1.02	–2.30
**[1-Ag]BAr$^F_4$**	+1.37	–0.54	–2.30
**[2-Ag]BAr$^F_4$**	+1.33	–0.54	–2.34

[a] Redox waves are irreversible. The half-peak potential is reported.[7] [b] Redox waves are reversible. [c] No clear oxidation was observed within the solvent's electrochemical window.

FT-IR spectra

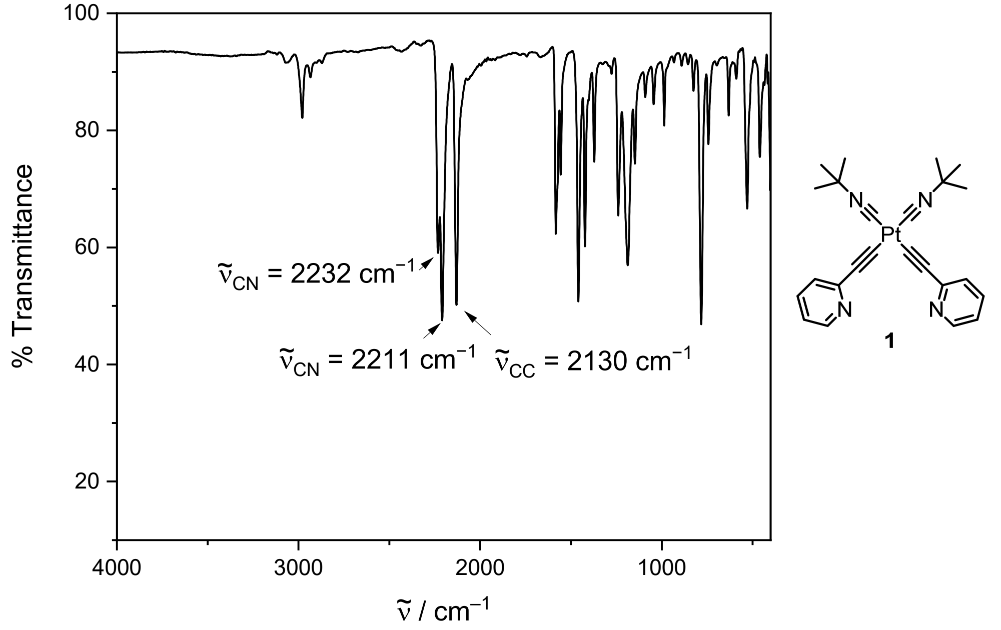

Fig. S16 FT-IR spectrum of complex **1**, recorded as a neat powder.

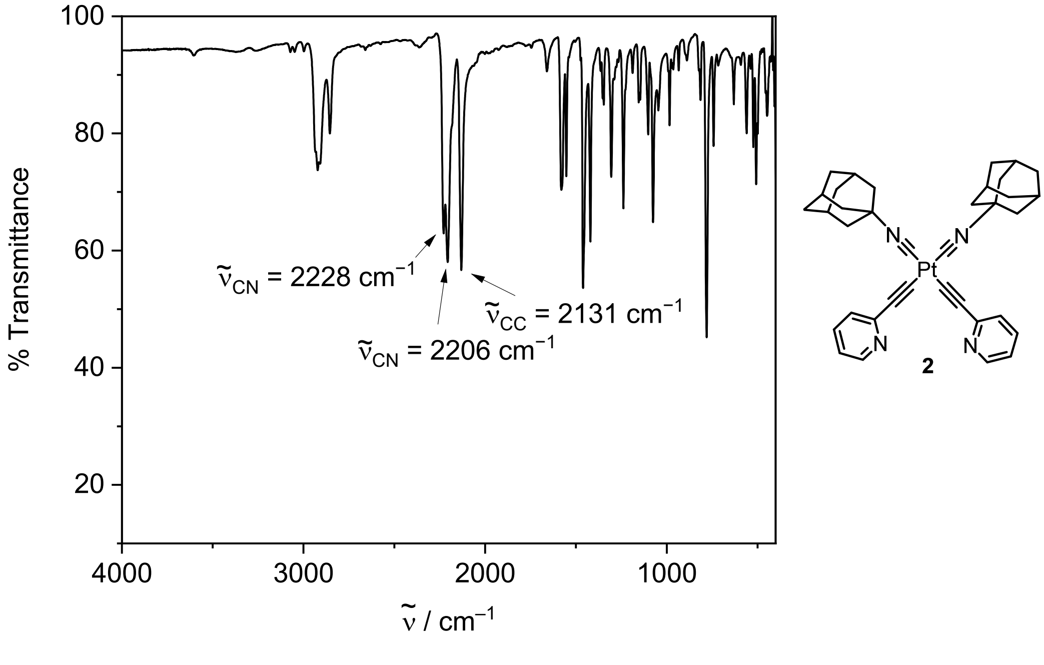

Fig. S17 FT-IR spectrum of complex **2**, recorded as a neat powder.

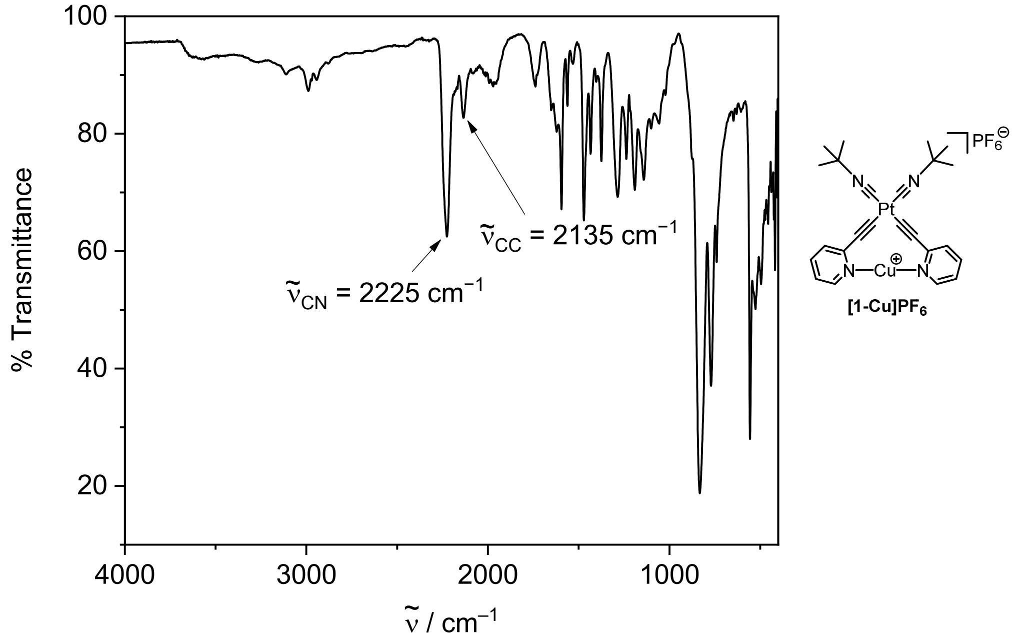

Fig. S18 FT-IR spectrum of complex **[1-Cu]PF$_6$**, recorded as a neat powder.

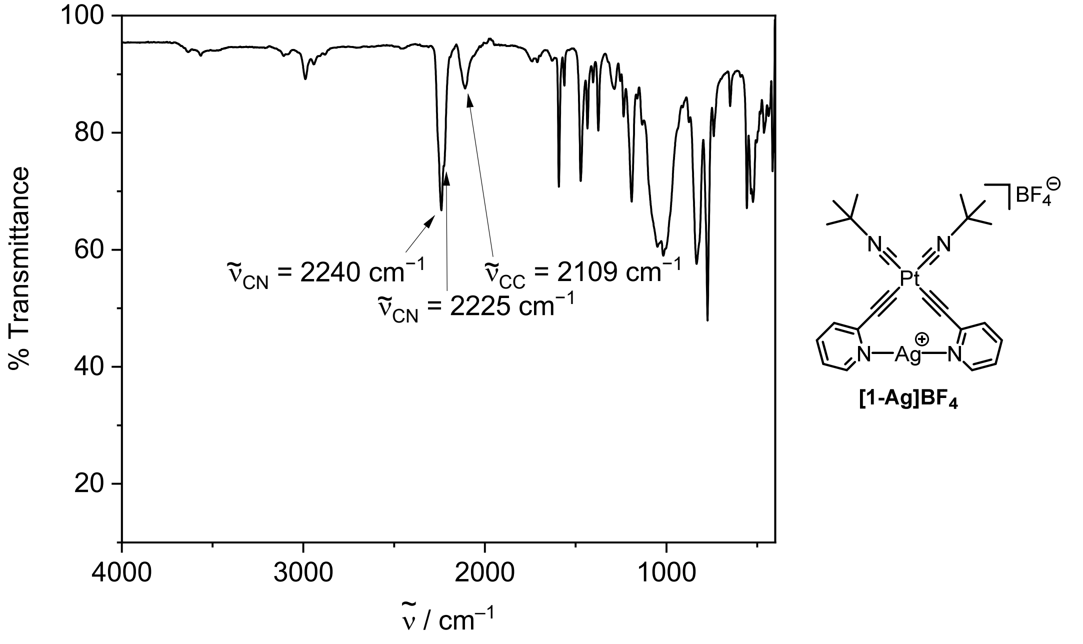

Fig. S19 FT-IR spectrum of complex **[1-Ag]BF$_4$**, recorded as a neat powder.

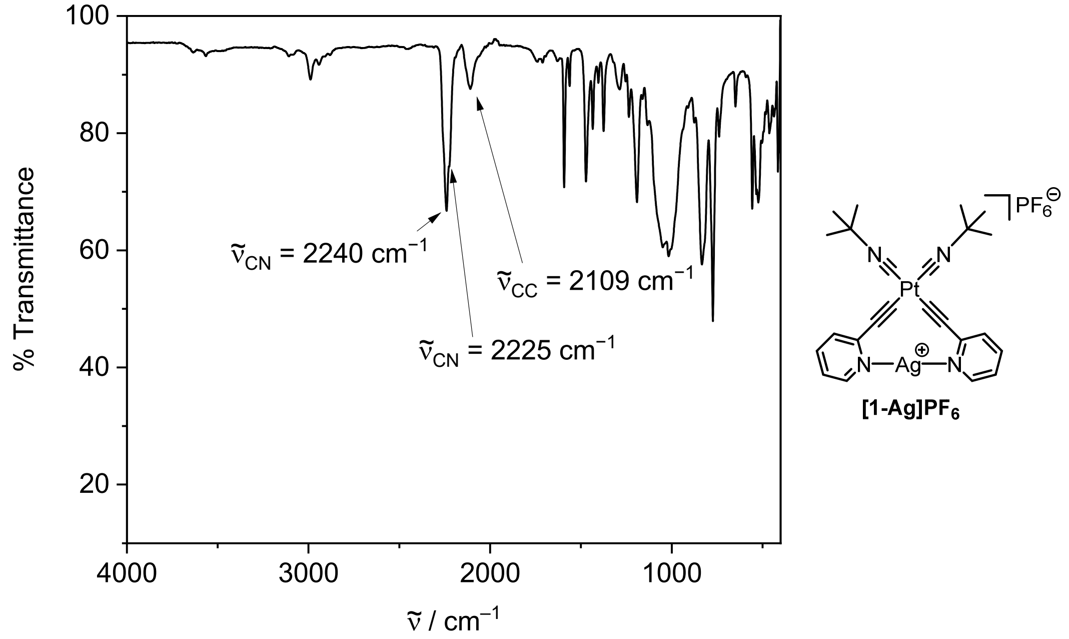

Fig. S20 FT-IR spectrum of complex **[1-Ag]PF$_6$**, recorded as a neat powder.

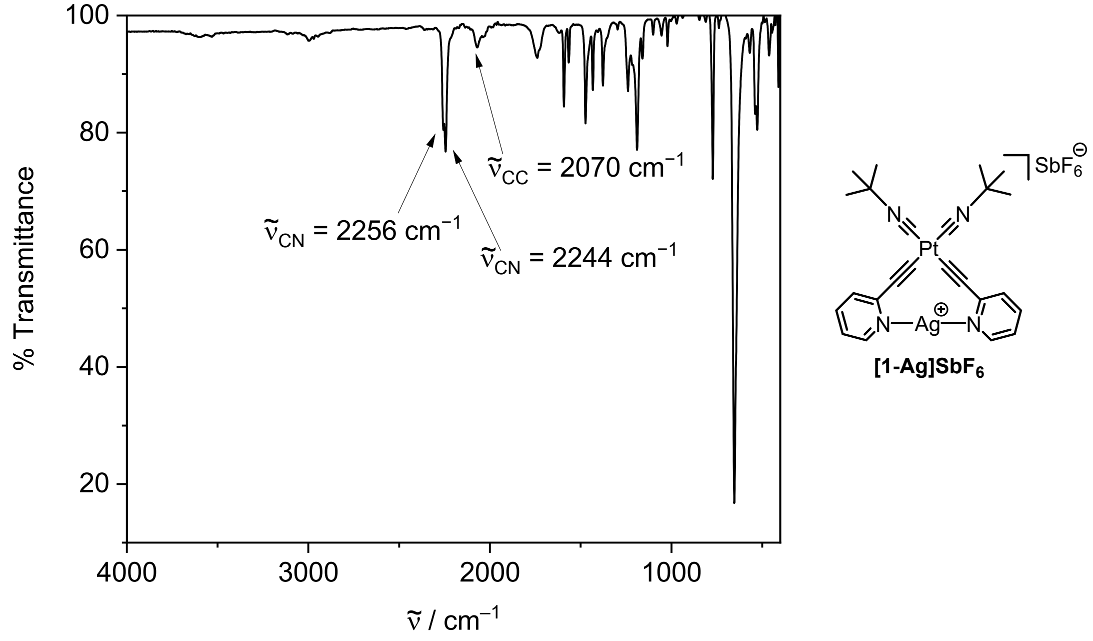

Fig. S21 FT-IR spectrum of complex **[1-Ag]SbF$_6$**, recorded as a neat powder.

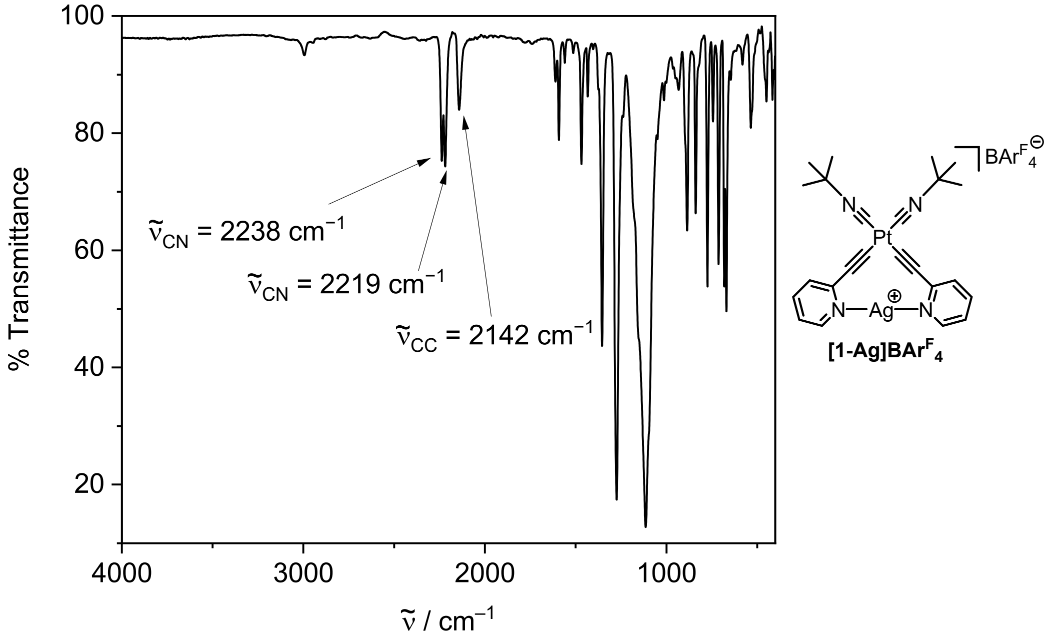

Fig. S22 FT-IR spectrum of complex **[1-Ag]BAr^F$_4$**, recorded as a neat powder.

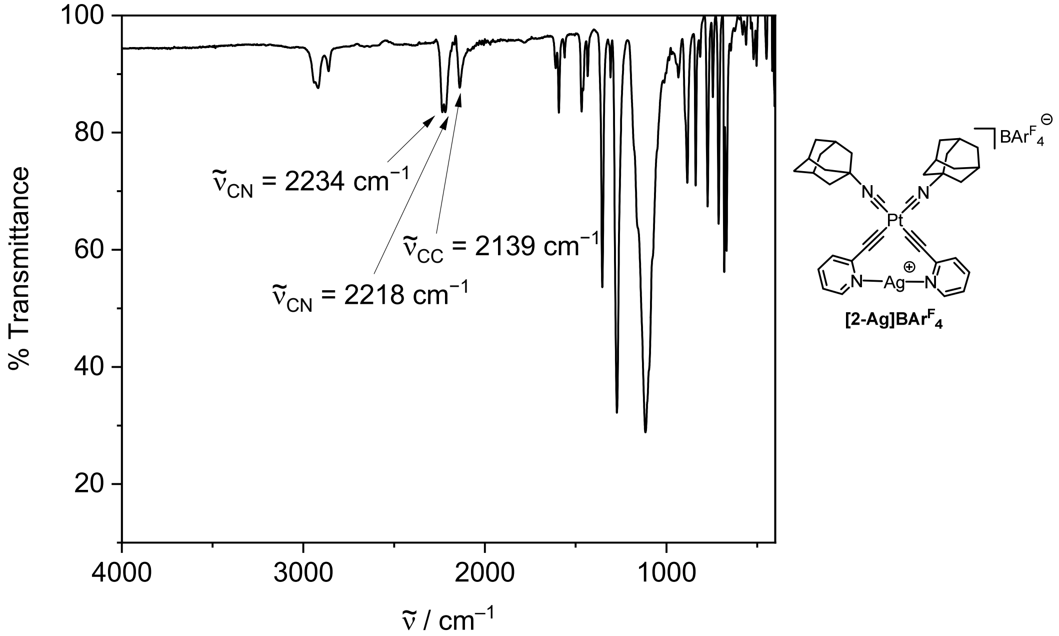

Fig. S23 FT-IR spectrum of complex **[2-Ag]BAr^F$_4$**, recorded as a neat powder.

NMR spectra

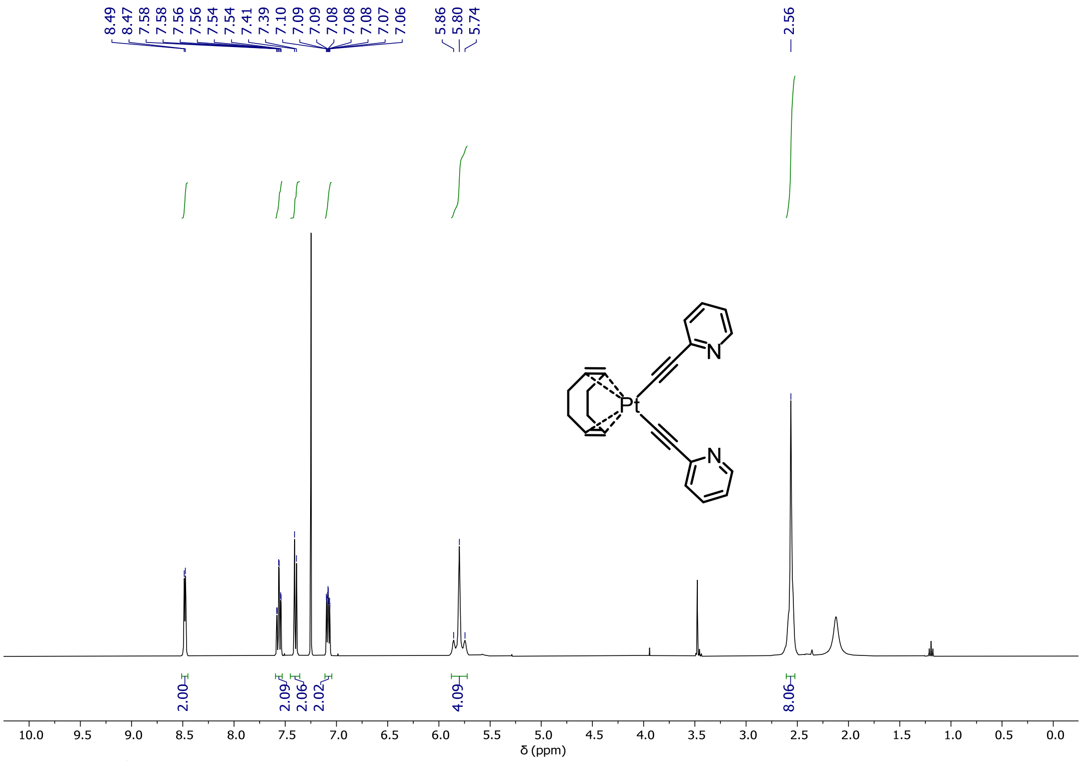

Fig. S24 ^{1}H NMR spectrum of [Pt(COD)(C≡C–2-py)$_2$], recorded in $CDCl_3$ at 400 MHz.

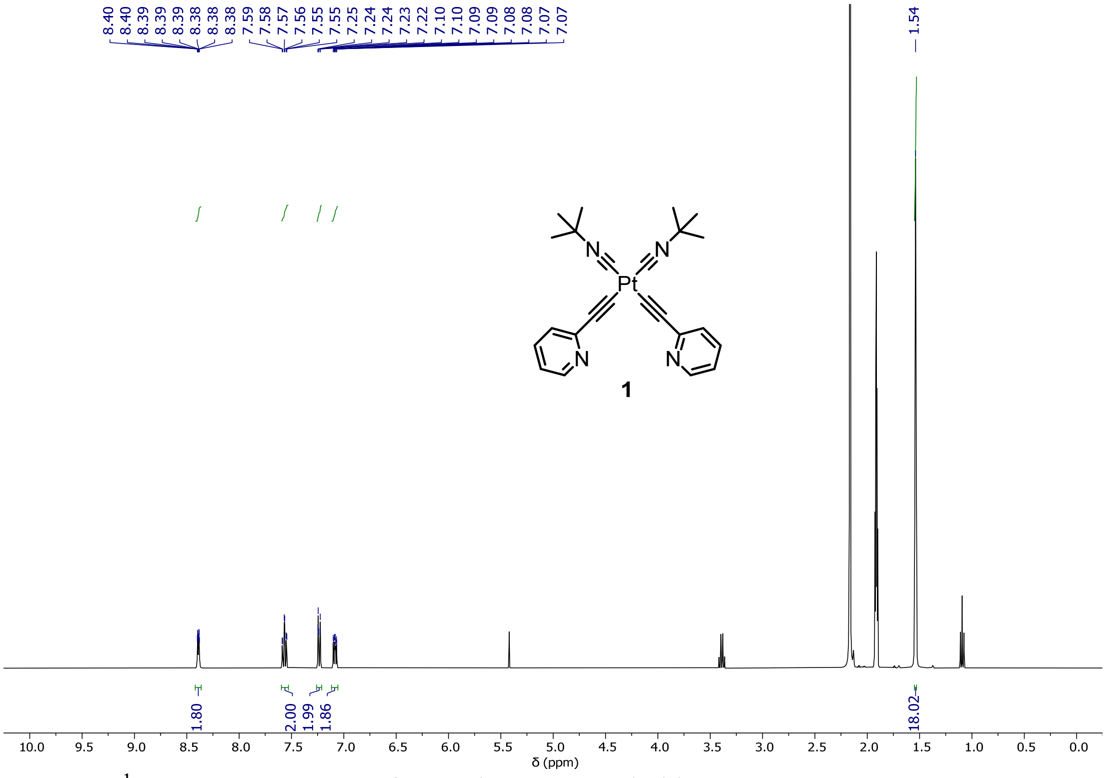

Fig. S25 ^{1}H NMR spectrum of complex **1**, recorded in CD_3CN at 400 MHz.

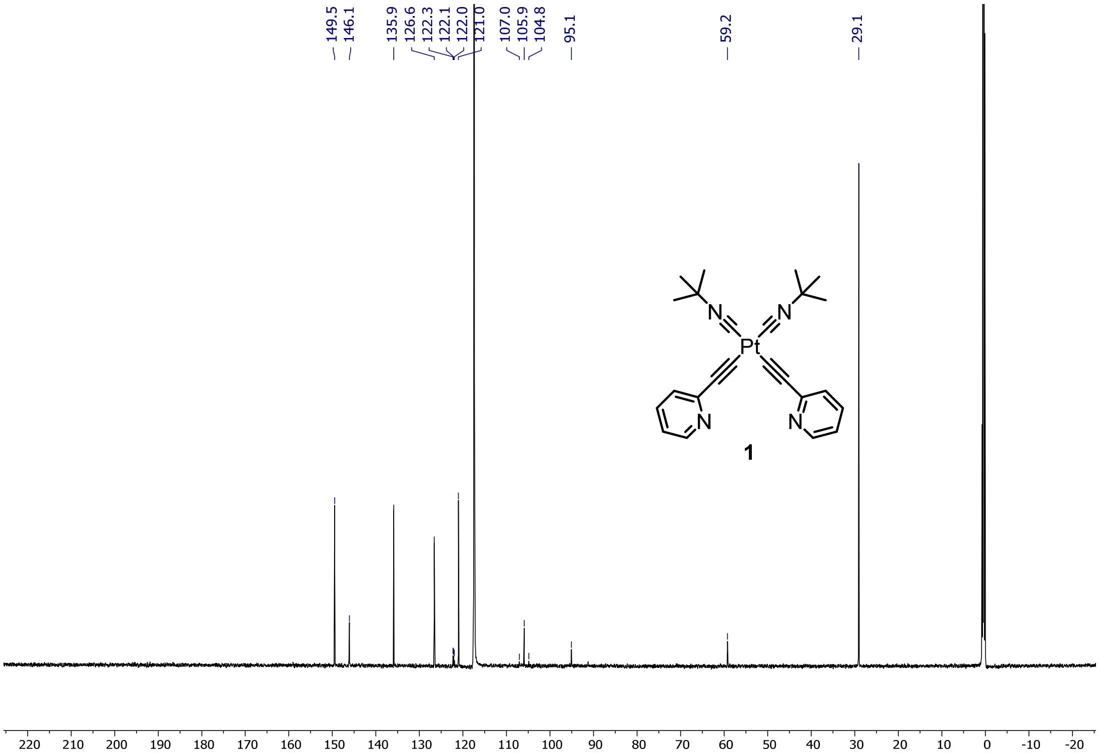

Fig. S26 $^{13}C\{^{1}H\}$ NMR spectrum of complex **1**, recorded in CD_3CN at 151 MHz.

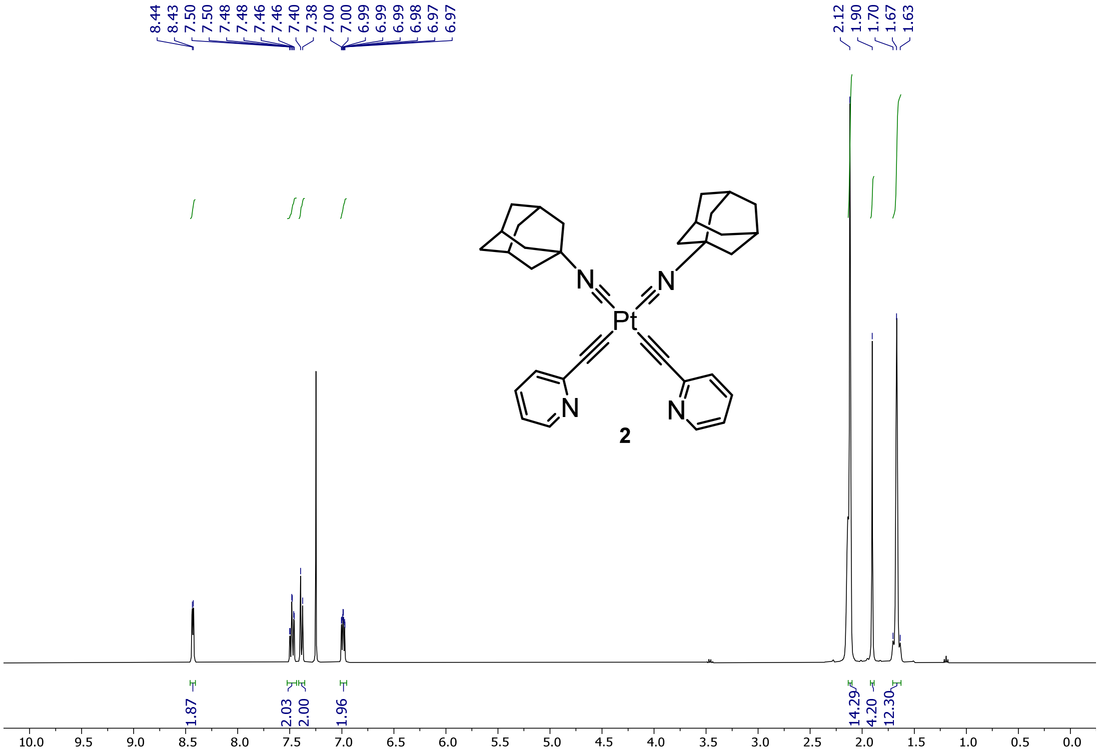

Fig. S27 ^{1}H NMR spectrum of complex **2**, recorded in $CDCl_3$ at 400 MHz.

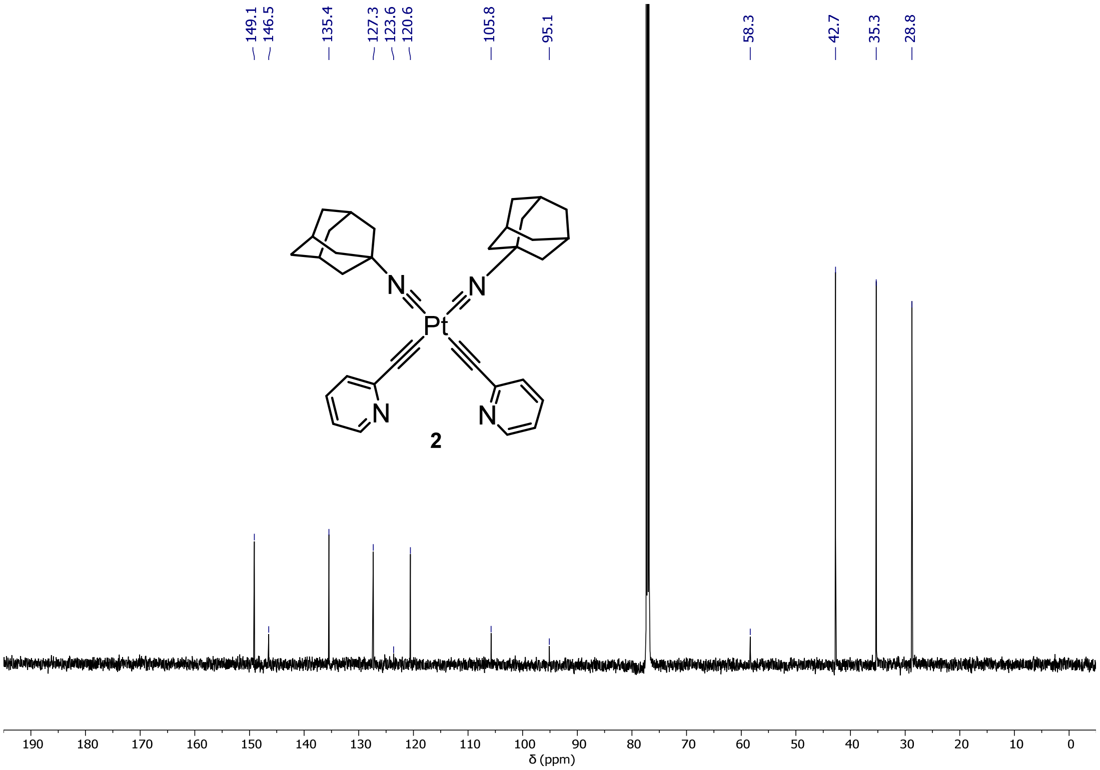

Fig. S28 $^{13}C\{^{1}H\}$ NMR spectrum of complex **2**, recorded in $CDCl_3$ at 151 MHz.

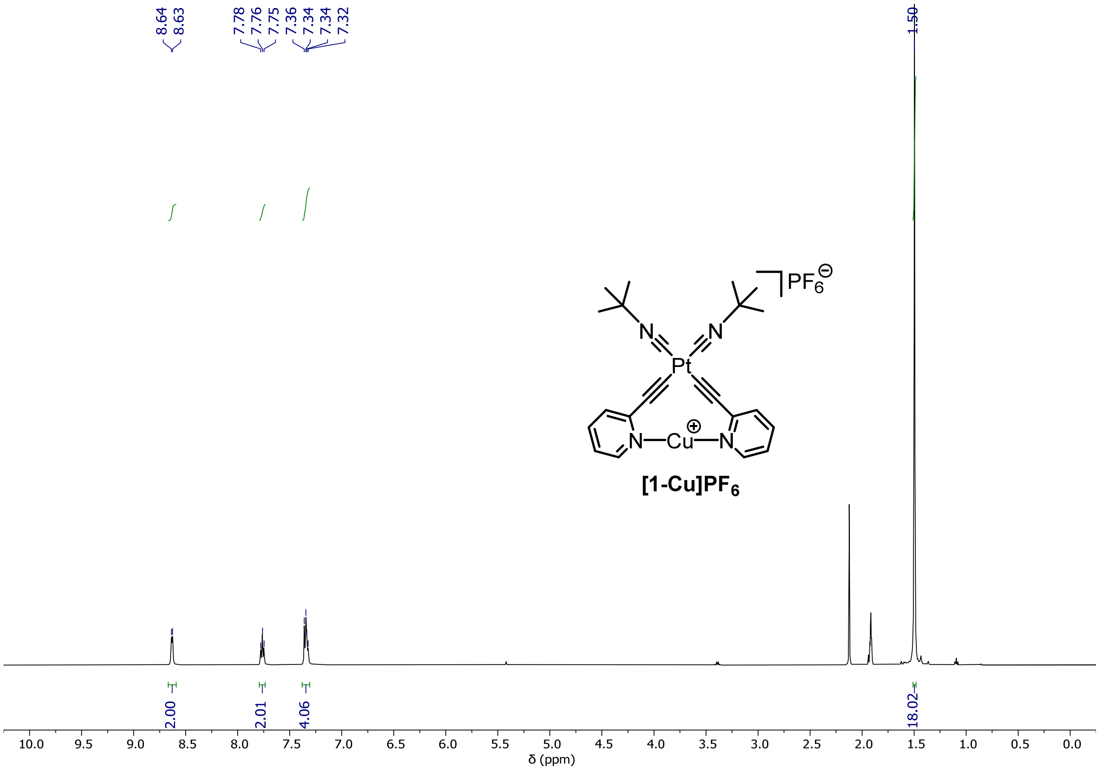

Fig. S29 ^{1}H NMR spectrum of complex **[1-Cu]PF$_6$**, recorded in CD_3CN at 500 MHz.

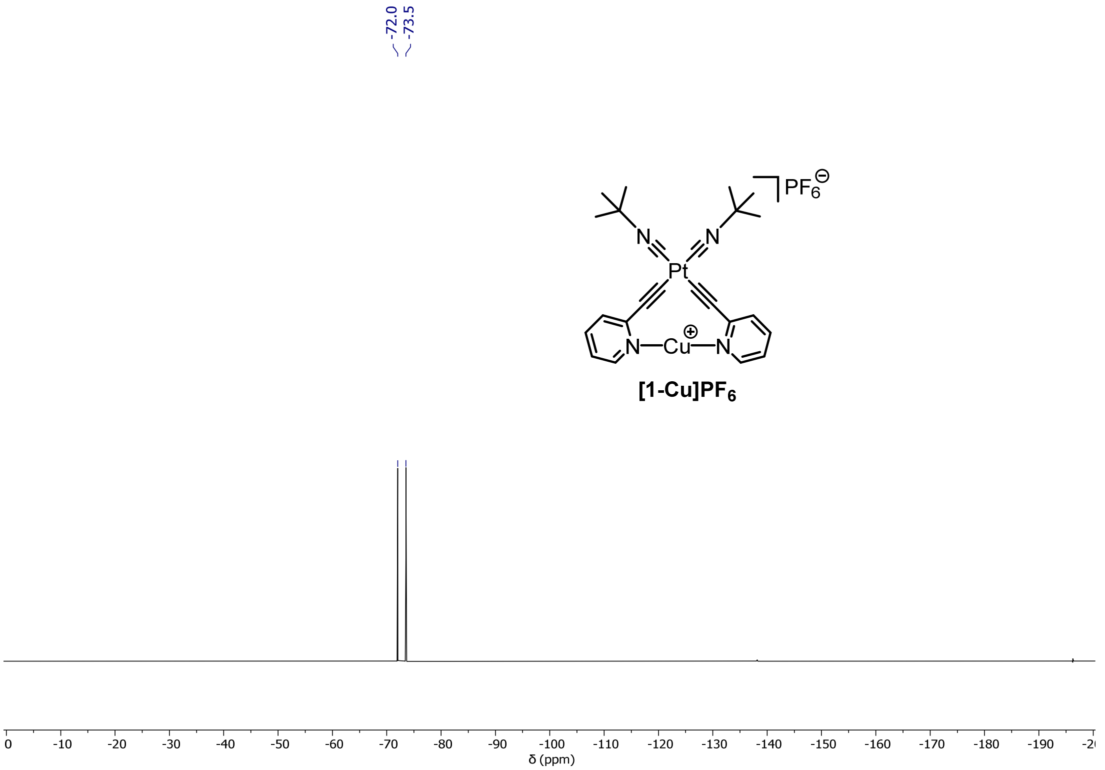

Fig. S30 ^{19}F NMR spectrum of complex **[1-Cu]PF$_6$**, recorded in CD_3CN at 470 MHz.

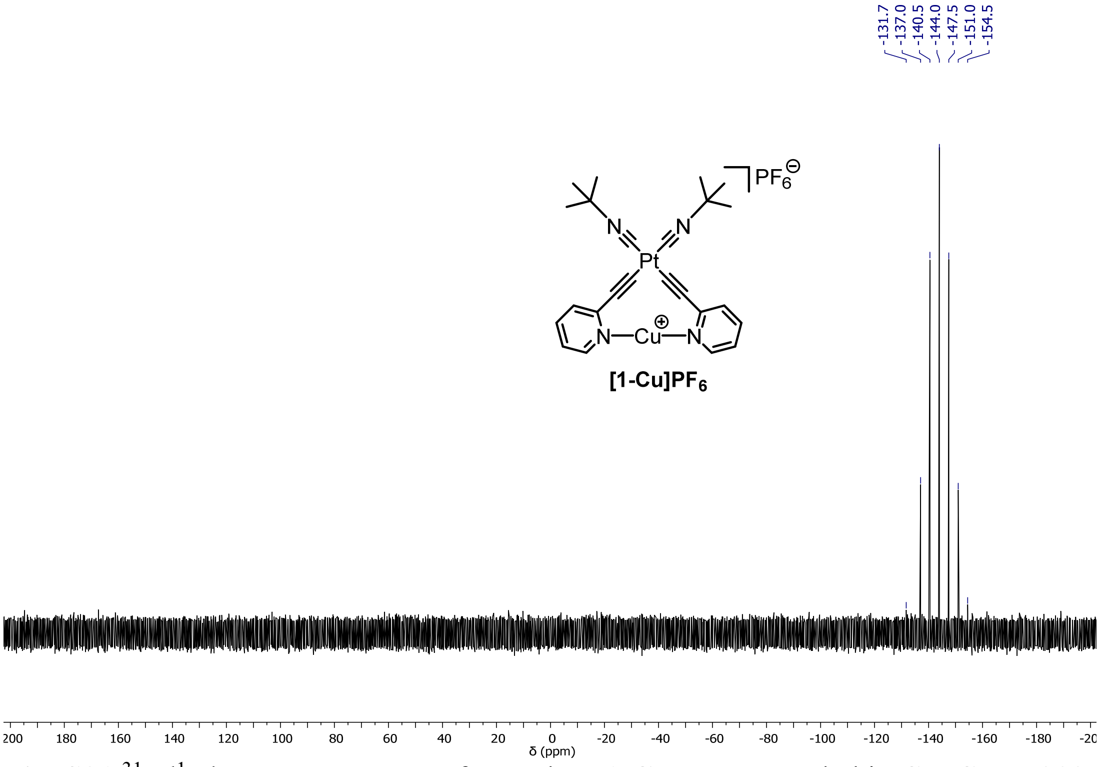

Fig. S31 $^{31}P\{^{1}H\}$ NMR spectrum of complex **[1-Cu]PF$_6$**, recorded in CD_3CN at 202 MHz.

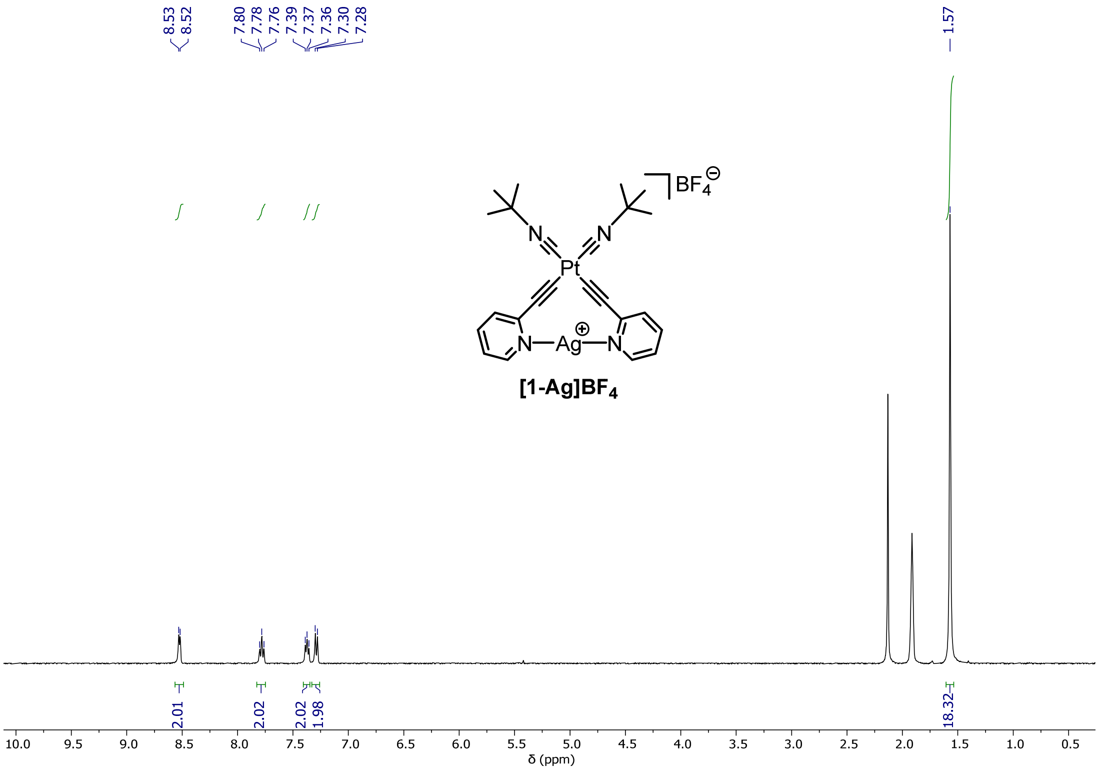

Fig. S32 ^{1}H NMR spectrum of complex **[1-Ag]BF$_4$**, recorded in CD_3CN at 400 MHz.

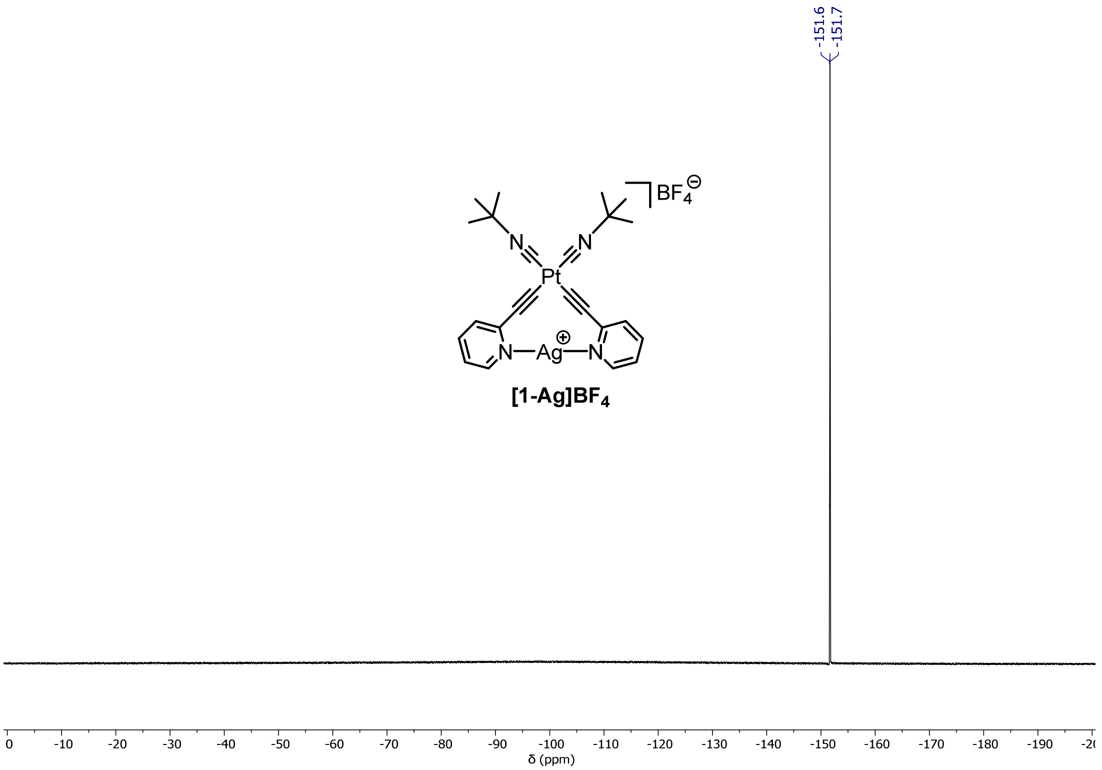

Fig. S33 ^{19}F NMR spectrum of complex **[1-Ag]BF$_4$**, recorded in CD_3CN at 376 MHz.

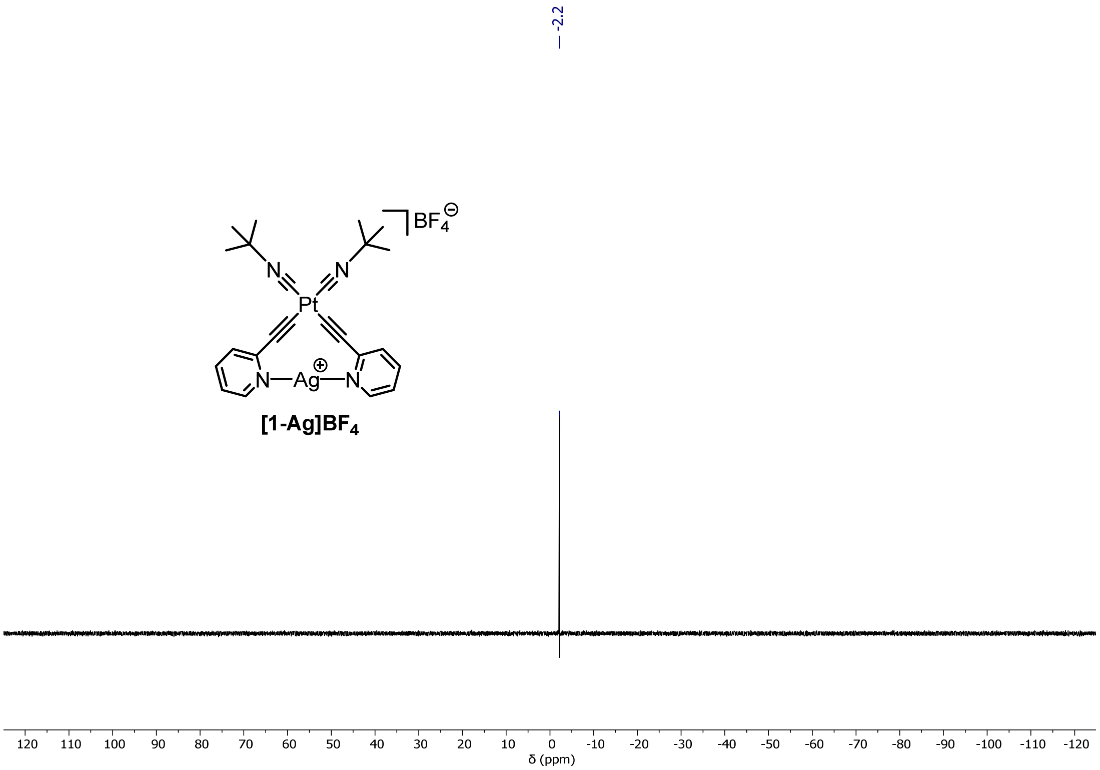

Fig. S34 $^{11}B\{^{1}H\}$ NMR spectrum of complex **[1-Ag]BF$_4$**, recorded in CD_3CN at 128 MHz.

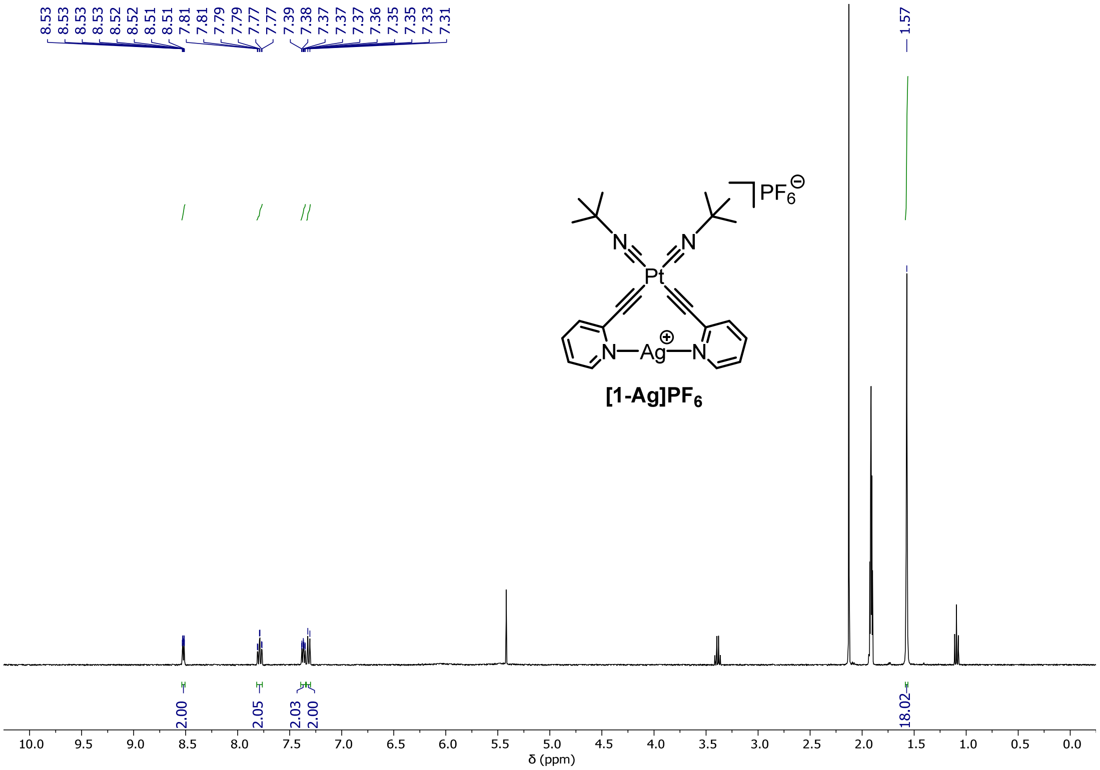

Fig. S35 ^{1}H NMR spectrum of complex **[1-Ag]PF$_6$**, recorded in CD_3CN at 400 MHz.

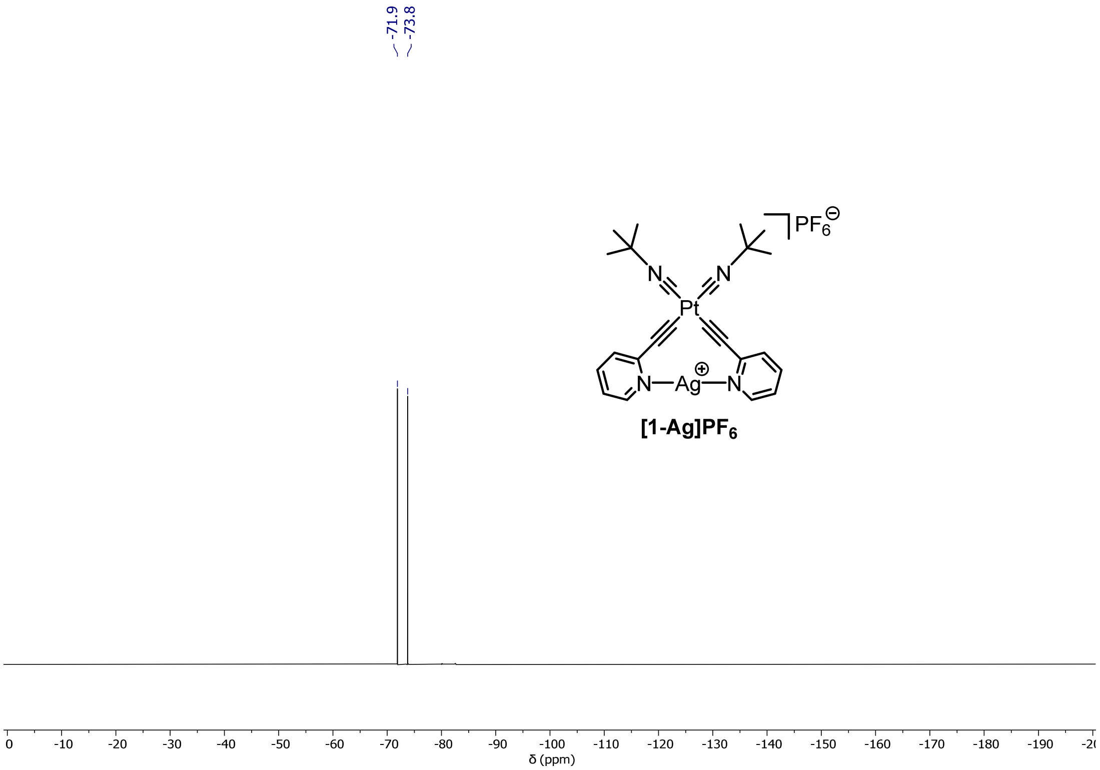

Fig. S36 ^{19}F NMR spectrum of complex **[1-Ag]PF$_6$**, recorded in CD_3CN at 470 MHz

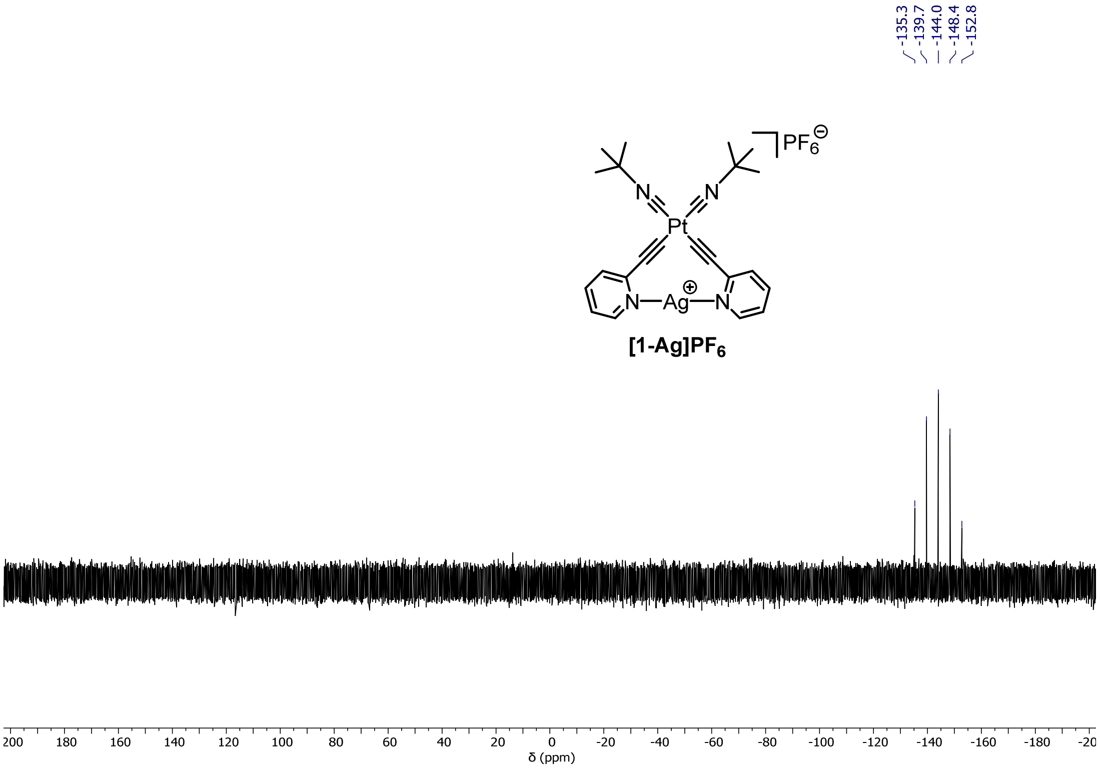

Fig. S37 ^{31}P{^{1}H} NMR spectrum of complex **[1-Ag]PF$_6$**, recorded in CD_3CN at 202 MHz.

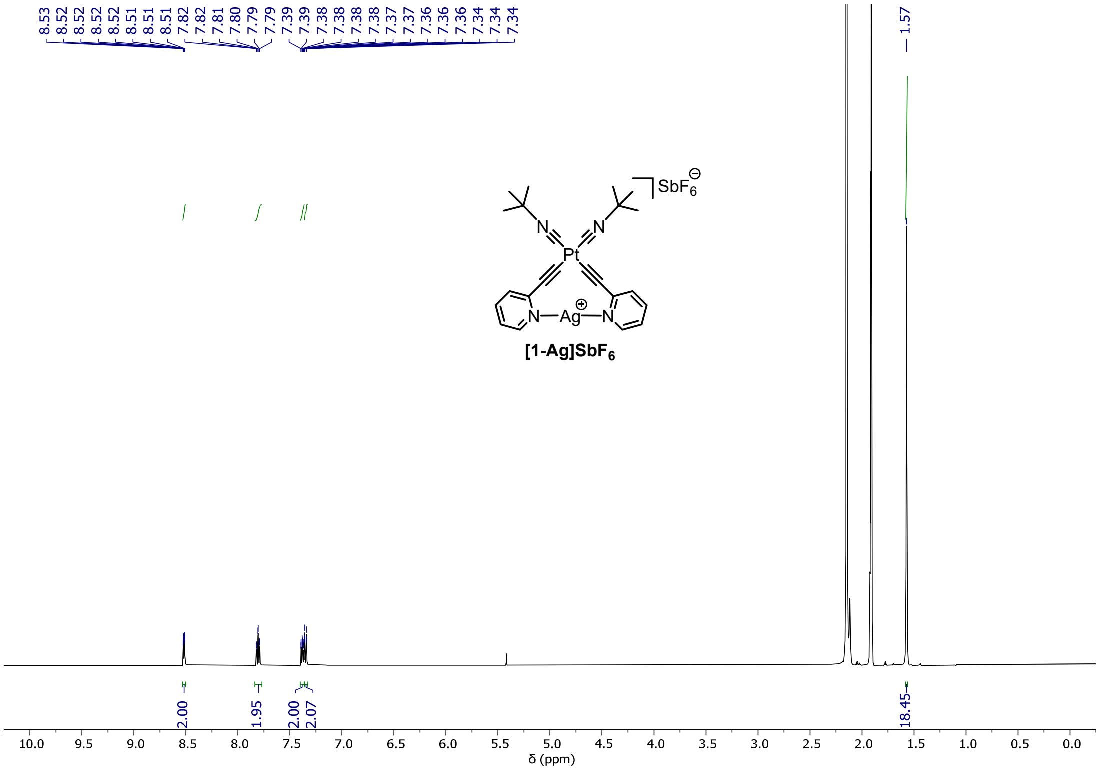

Fig. S38 ^{1}H NMR spectrum of complex **[1-Ag]SbF$_6$**, recorded in CD_3CN at 500 MHz.

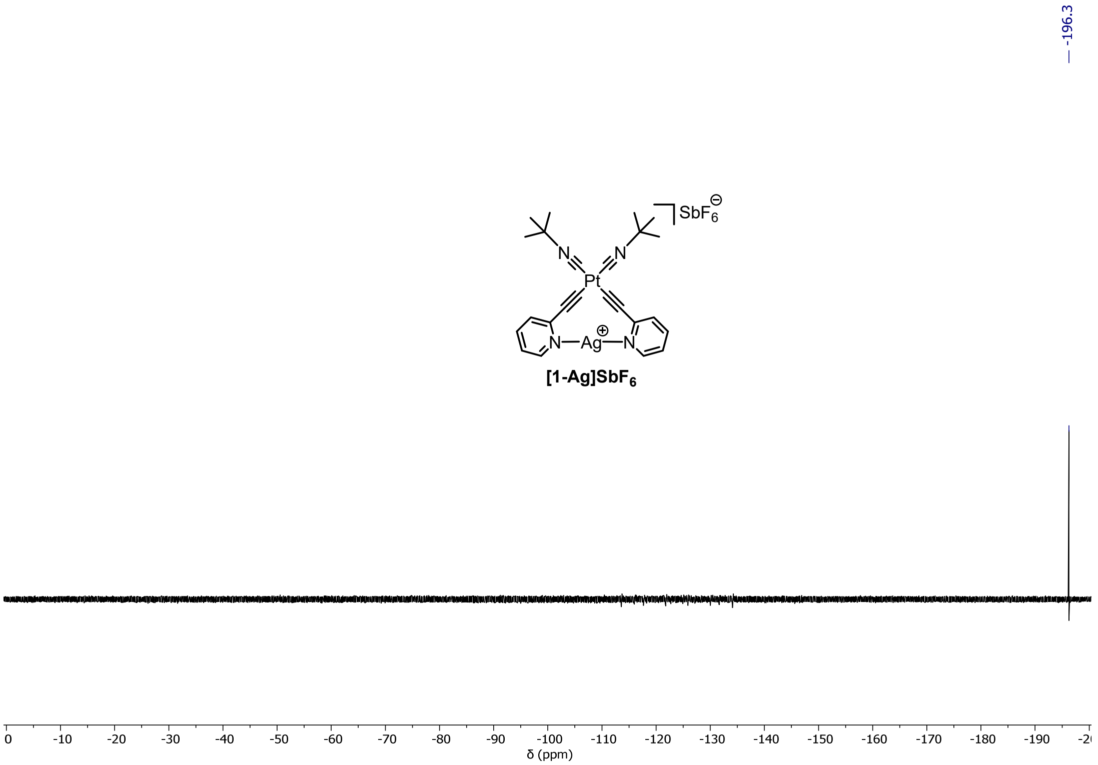

Fig. S39 ^{19}F NMR spectrum of complex **[1-Ag]SbF$_6$**, recorded in CD_3CN at 470 MHz.

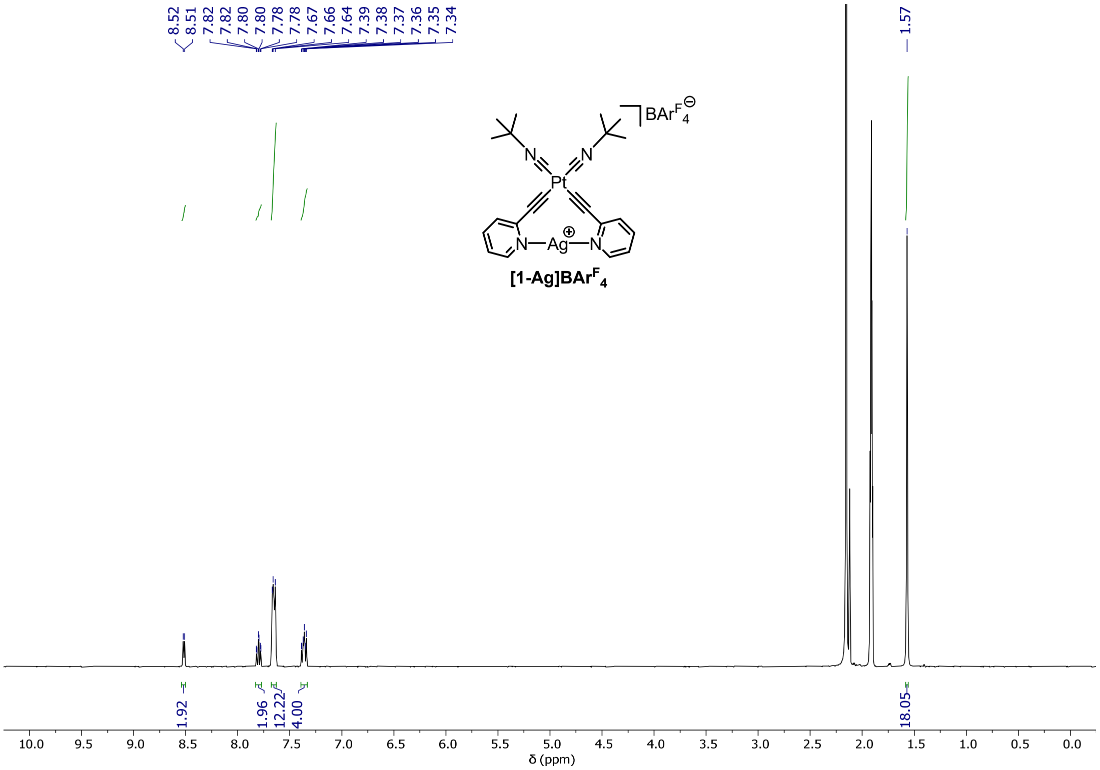

Fig. S40 ^{1}H NMR spectrum of complex **[1-Ag]BAr$^{F}_{4}$**, recorded in CD_3CN at 400 MHz.

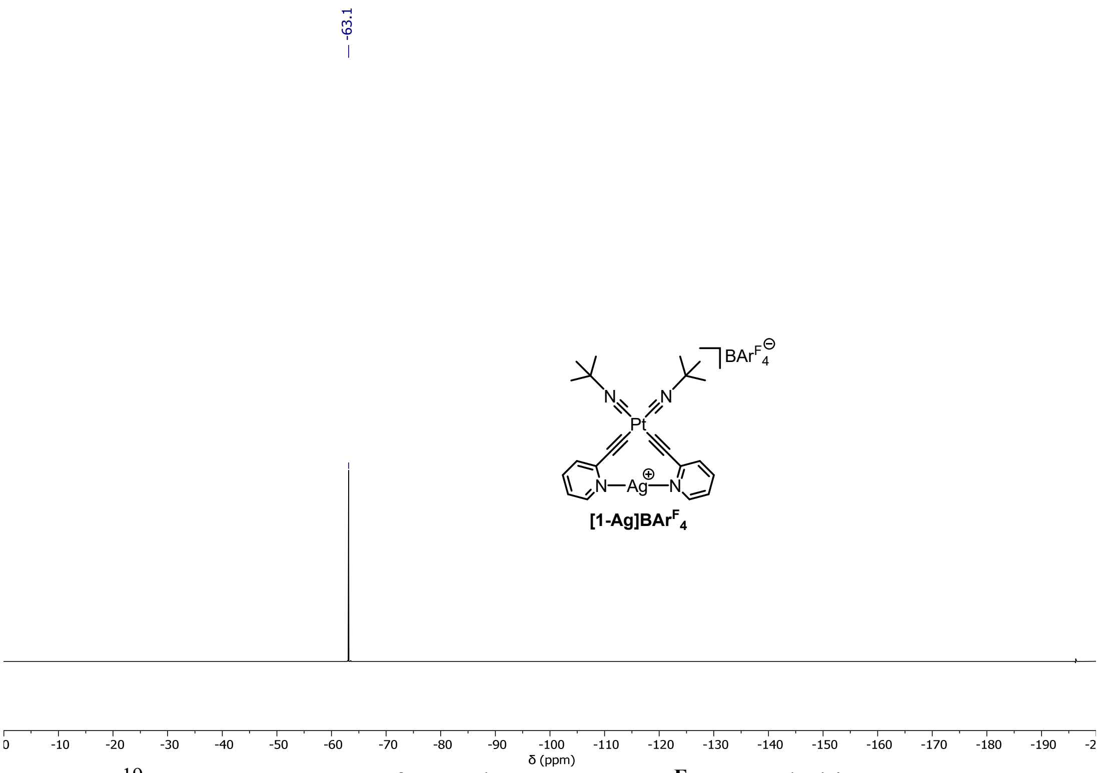

Fig. S41 ^{19}F NMR spectrum of complex **[1-Ag]BAr$^{F}_{4}$**, recorded in CD_3CN at 470 MHz

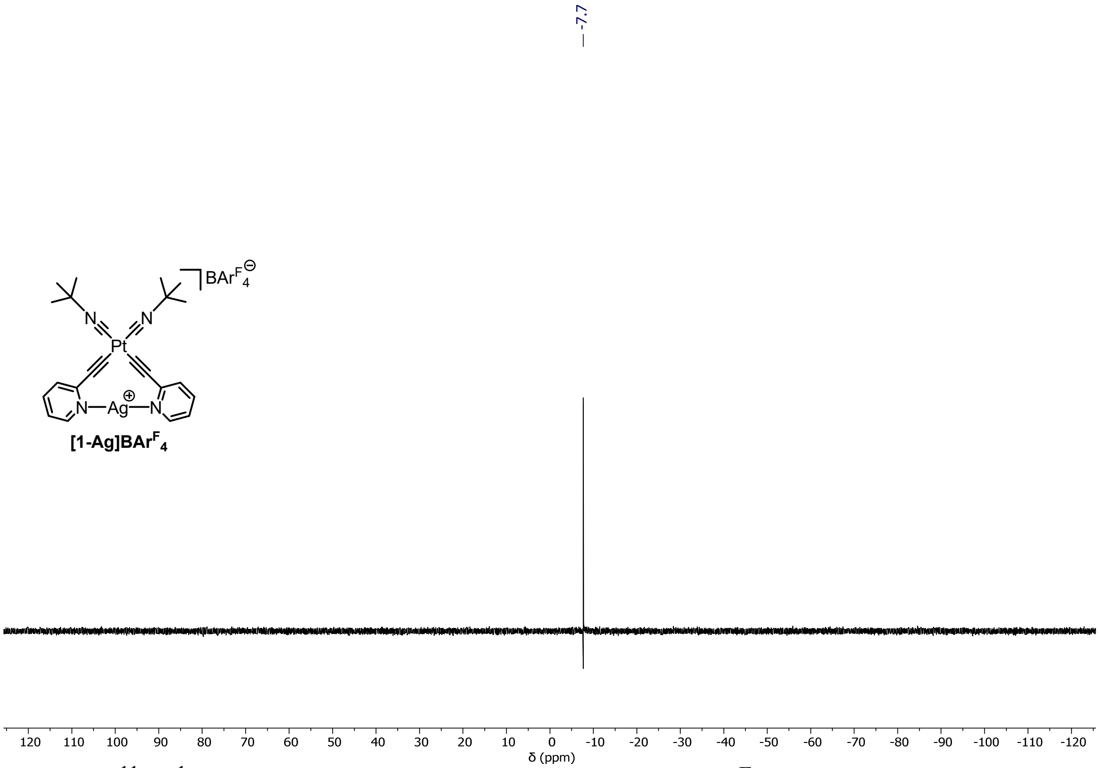

Fig. S42 $^{11}B\{^1H\}$ NMR spectrum of complex **[1-Ag]BAr$^F_4$**, recorded in CD_3CN at 160 MHz.

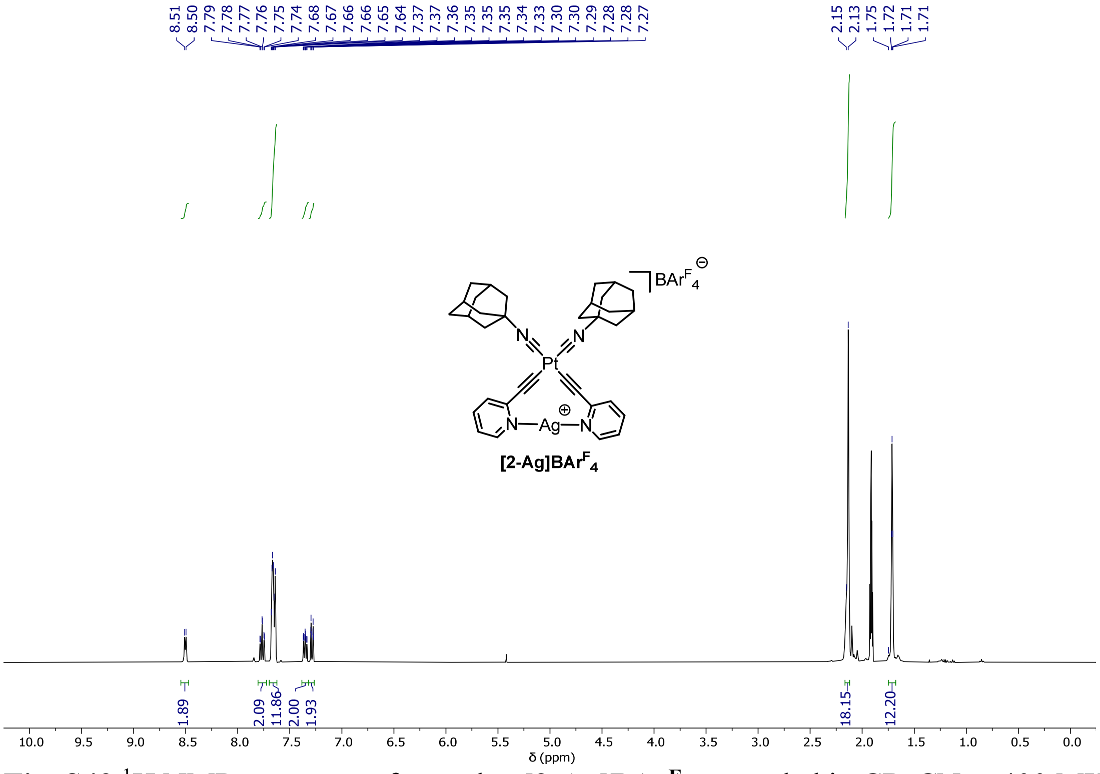

Fig. S43 ^1H NMR spectrum of complex **[2-Ag]BAr$^F_4$**, recorded in CD_3CN at 400 MHz.

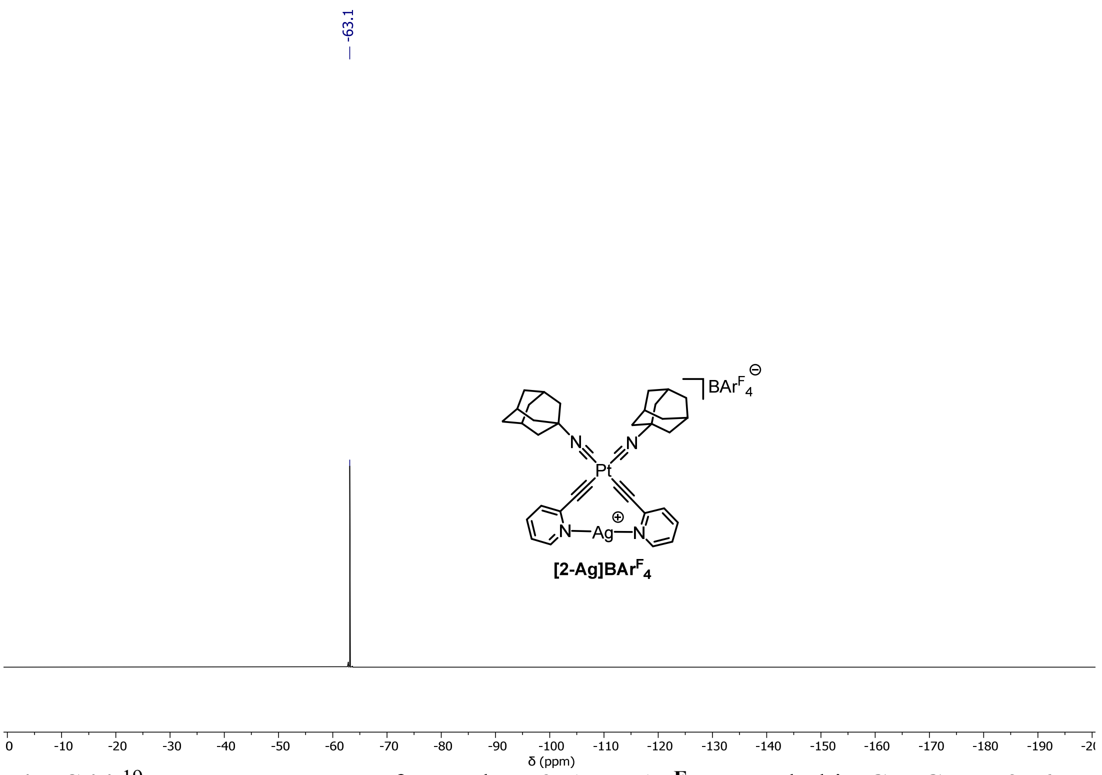

Fig. S44 ^{19}F NMR spectrum of complex **[2-Ag]BAr^{F}**$_4$, recorded in CD_3CN at 376 MHz.

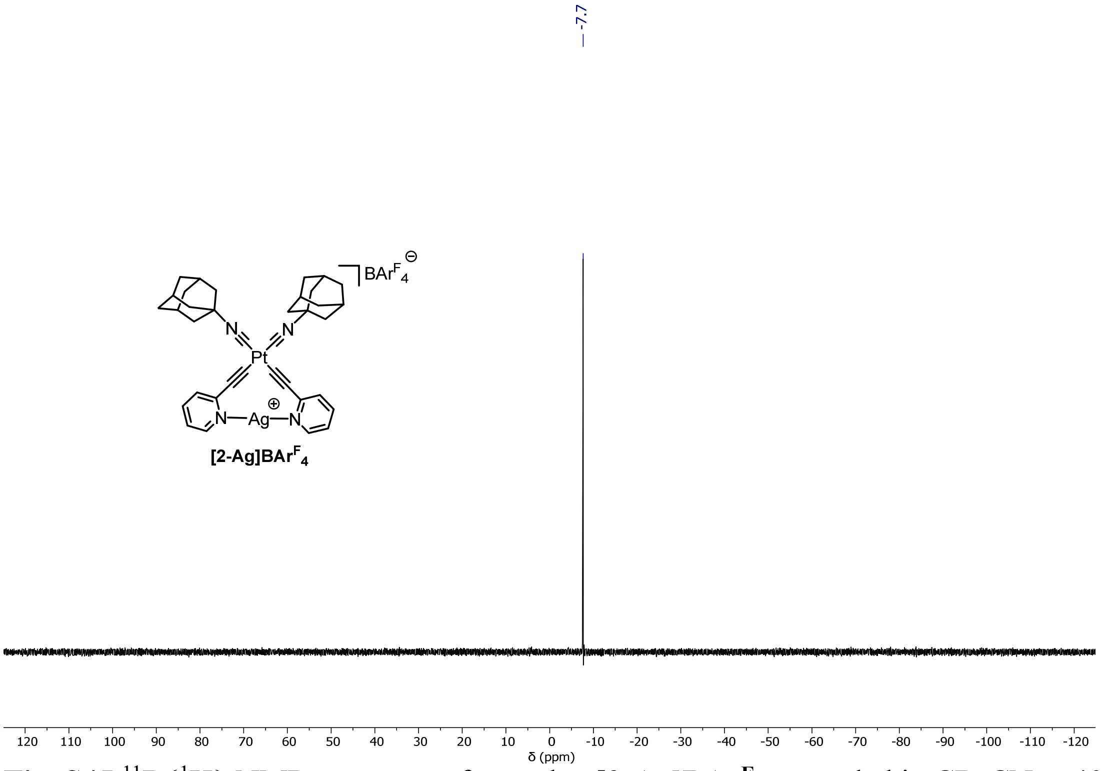

Fig. S45 ^{11}B{^{1}H} NMR spectrum of complex [**2-Ag]BAr^{F}**$_4$, recorded in CD_3CN at 400 MHz.

References

1 J. X. McDermott, J. F. White and G. M. Whitesides, *J. Am. Chem. Soc.*, 1976, **98**, 6521–6528.

2 N. Hidalgo, J. J. Moreno, I. García-Rubio and J. Campos, *Angew. Chem. Int. Ed.*, 2022, **61**, e202206831.

3 K. Suzuki, A. Kobayashi, S. Kaneko, K. Takehira, T. Yoshihara, H. Ishida, Y. Shiina, S. Oishi and S. Tobita, *Phys. Chem. Chem. Phys.*, 2009, **11**, 9850.

4 A. Lüning, J. Schur, L. Hamel, I. Ott and A. Klein, *Organometallics*, 2013, **32**, 3662–3672.

5 Y. Wu, Z. Wen, J. I. Wu and T. S. Teets, *Chem. – Eur. J.*, 2020, **26**, 16028–16035.

6 Y. H. Nguyen, J. V. Soares, S. H. Nguyen, Y. Wu, J. I. Wu and T. S. Teets, *Inorg. Chem.*, 2022, **61**, 8498–8508.

7 E. M. Espinoza, J. A. Clark, J. Soliman, J. B. Derr, M. Morales and V. I. Vullev, *J. Electrochem. Soc.*, 2019, **166**, H3175–H3187.
